# The similar and different evolutionary trends of MATE family occurred between rice and *Arabidopsis thaliana*

**DOI:** 10.1186/s12870-016-0895-0

**Published:** 2016-09-26

**Authors:** Lihui Wang, Xiujuan Bei, Jiansheng Gao, Yaxuan Li, Yueming Yan, Yingkao Hu

**Affiliations:** College of Life Sciences, Capital Normal University, Beijing, 100048 China

**Keywords:** MATE proteins, Phylogenetic tree, Segmental duplication, Tandem duplication, Functional divergence, Positive selection

## Abstract

**Background:**

Multidrug and toxic compound extrusion (MATE) transporter proteins are present in all organisms. Although the functions of some MATE gene family members have been studied in plants, few studies have investigated the gene expansion patterns, functional divergence, or the effects of positive selection.

**Results:**

Forty-five MATE genes from rice and 56 from Arabidopsis were identified and grouped into four subfamilies. MATE family genes have similar exon-intron structures in rice and Arabidopsis; MATE gene structures are conserved in each subfamily but differ among subfamilies. In both species, the MATE gene family has expanded mainly through tandem and segmental duplications. A transcriptome atlas showed considerable differences in expression among the genes, in terms of transcript abundance and expression patterns under normal growth conditions, indicating wide functional divergence in this family. In both rice and Arabidopsis, the MATE genes showed consistent functional divergence trends, with highly significant Type-I divergence in each subfamily, while Type-II divergence mainly occurred in subfamily III. The Type-II coefficients between rice subfamilies I/III, II/III, and IV/III were all significantly greater than zero, while only the Type-II coefficient between Arabidopsis IV/III subfamilies was significantly greater than zero.

A site-specific model analysis indicated that MATE genes have relatively conserved evolutionary trends. A branch-site model suggested that the extent of positive selection on each subfamily of rice and Arabidopsis was different: subfamily II of Arabidopsis showed higher positive selection than other subfamilies, whereas in rice, positive selection was highest in subfamily III. In addition, the analyses identified 18 rice sites and 7 Arabidopsis sites that were responsible for positive selection and for Type-I and Type-II functional divergence; there were no common sites between rice and Arabidopsis. Five coevolving amino acid sites were identified in rice and three in Arabidopsis; these sites might have important roles in maintaining local structural stability and protein functional domains.

**Conclusions:**

We demonstrate that the MATE gene family expanded through tandem and segmental duplication in both rice and Arabidopsis. Overall, the results of our analyses contribute to improved understanding of the molecular evolution and functions of the MATE gene family in plants.

**Electronic supplementary material:**

The online version of this article (doi:10.1186/s12870-016-0895-0) contains supplementary material, which is available to authorized users.

## Background

Plants are routinely exposed to exogenous toxins secreted by other organisms or pathogenic microbes and to endogenous toxins produced by metabolic processes. Thus, disposal and detoxification of toxic compounds of both exogenous and endogenous origin are important processes for survival and development. There are several possible mechanisms for detoxification: modification of the toxic compounds by endogenous enzymes [1, 2]; target alteration [3]; sequestration into the vacuole [4–7]; and, transport outside of the cell [8, 9]. Integral membrane proteins named ‘multidrug resistance transporter’ are important drug resistance pumps as they can extrude structurally and chemically distinct drugs from cells, giving rise to multidrug resistance [10, 11]. Multidrug transporters are classified into five main groups [8, 12]: ATP-binding cassette (ABC), major facilitator superfamily (MFS), resistance-nodulation-division (RND), small multidrug resistance (SMR) transporters, and multidrug and toxic compound extrusion (MATE) families. The primary ABC transporters use the energy of ATP hydrolysis to transport drugs, whereas the other families are secondary transporters that use H^+^ or Na^+^ electrochemical gradients to drive substrate export.

Multidrug and toxic compound extrusion (MATE) proteins are widely present in bacteria, fungi, plants, and mammals. Most members of the MATE family consist of 440–550 amino acids with 12 transmembrane helices, although they can range from ~400 to ~700 residues. MATE proteins do not appear to have a conserved consensus sequence; however, all MATE proteins share ~40 % sequence similarity. In contrast to the bacterial and animal kingdoms, which have a relatively small number of MATE genes per species, plants contain many MATE-type transporters. For instance, *Arabidopsis thaliana* possesses 58 MATE orthologs, although their transport properties have not all been elucidated [13]. In rice, a search of the genome database indicated that there are at least 53 MATE genes [14].

Previous studies have shown that MATE proteins in plants have various functions. For example, a defect in the Arabidopsis *ALF5* gene arrests root growth in plants grown on agar, probably owing to increased sensitivity to unidentified soluble contaminants [15]. The *ALF5* gene product is presumed to be present in the vacuoles of the root epidermis, while expression of *ALF5* in yeast confers resistance to tetraethylammonium (TEA) [15]. The Arabidopsis transparent testa 12 (*tt12*) gene also encodes a MATE-type transporter [16, 17], which acts as a vacuolar flavonoid/H+ − antiporter active in proanthocyanidin-accumulating cells of the seed coat and facilitates vacuolar uptake of epicatechin 3'-O-glucoside for proanthocyanidin biosynthesis in *Medicago truncatula* and Arabidopsis [18, 19]. A similar MATE transporter has been identified in tomato [20]. The Arabidopsis MATE transporter DTX1 is localized in the plasma membrane and mediates the export of exogenous toxic compounds such as TEA and berberine [21]. The MATE genes *HvAACT1* and SbMATE are involved in aluminum tolerance in barley and sorghum, respectively [22, 23]. FRD3 from Arabidopsis has been demonstrated to be a citrate transporter, and is required for Fe transportation from the roots to the shoot [24, 25]. Analysis of the rice MATE gene *OsFRDL1*, which is the closest homolog of barley *HvAACT1*, indicated that it encodes a protein that is localized in pericyclic cells and acts as a citrate transporter, which is necessary for the efficient translocation of Fe to the shoot as an Fe-citrate complex [26].

Although the functions of MATE gene family members have been resolved in different species, investigation of this gene family from a genomics viewpoint has not been performed. In the present study, all the MATE protein-encoding sequences members were identified from rice, a monocot species, and Arabidopsis, a dicot species. Phylogenetic analysis, examination of exon-intron structures, and gene expansion patterns analysis were performed to explore the similarities and differences in the MATE gene family of these two species. We also analyzed the expression profiles of MATE genes in different tissues of rice and Arabidopsis. To determine whether there was a similar driving force for the evolution of function in rice and Arabidopsis, we analyzed functional divergence and adaptive evolution in the two species. In addition, a coevolution analysis was performed to identify instances of coevolution between amino acid sites in rice and Arabidopsis.

## Results

### Genome-wide identification of the MATE gene family in rice and Arabidopsis

**Fig. 1:**
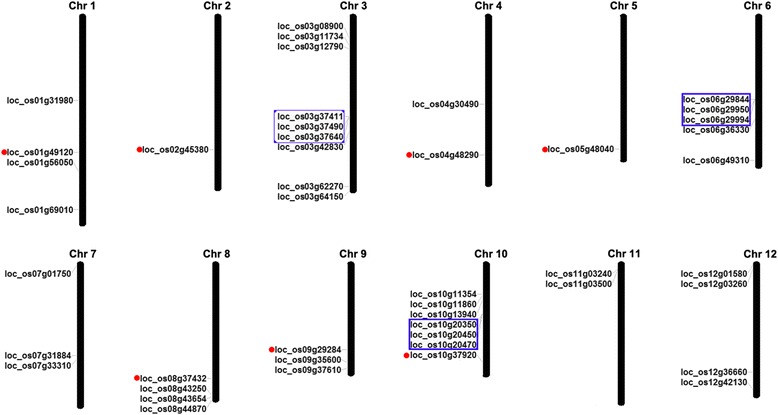
Chromosomal distribution of rice MATE genes. Chromosome sizes are indicated by relative lengths. Tandemly duplicated genes are indicated by the boxes with blue outlines. Segmentally duplicated genes are indicated by the red dots to the left. The figure was produced using the Map Inspector program

**Fig. 2:**
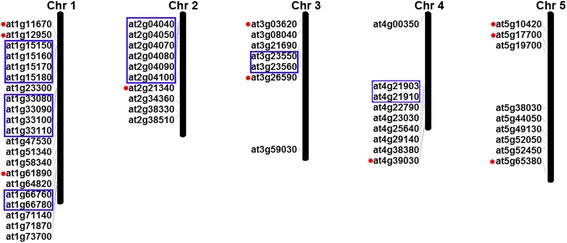
Chromosomal distribution of *Arabidopsis thaliana* MATE genes. Chromosome sizes are indicated by relative lengths. Tandemly duplicated genes are indicated by the boxes with blue outlines. Segmentally duplicated genes are indicated by the red dots to the left. The figure was produced using the Map Inspector program

The two plant species selected here, the monocot *Oryza sativa* and the dicot *Arabidopsis thaliana*, represent model organisms for the two major plant lineages. A BLASTP search of the Phytozome database (https://phytozome.jgi.doe.gov/pz/portal.html) identified 45 MATE genes in *Oryza sativa* and 56 in *Arabidopsis thaliana*. Both PFAM and SMART databases confirmed the presence of the conserved domain in the MATE gene family. The protein sequences (Additional file 1), coding sequences (Additional file 2), and genomic sequences (Additional file 3) were all obtained from the Phytozome database. Basic information on the rice and Arabidopsis MATE genes (including gene name, locus, protein length, intron number, PI value, and molecular weight) is provided in Additional files 4 and 5. The 45 MATE rice genes encoded proteins of 392 to 644 amino acids, with molecular weights ranging from 41.3 to 65.8 kD, and pI values from 5.14 to 10.07. Likewise, the 56 Arabidopsis genes encoded proteins with amino acid sequence lengths of 469 to 575 amino acids, molecular weights from 50.8 to 63.5 kD, and pI values ranging from 4.66 to 8.67. These results implied that the amino acid sequence length and physicochemical properties of rice and Arabidopsis MATE proteins might have changed to meet different functions. The genes for the rice and Arabidopsis MATE proteins were mapped to their chromosomes (Figs. 1 and 2). In Arabidopsis, the predicted 56 AtMATE (*Arabidopsis thaliana* MATE protein) genes were located on five chromosomes. Chromosome 1 had 21 AtMATE genes, while 10, 7, 9, and 9 AtMATE genes were found on chromosomes 2, 3, 4, and 5, respectively. In rice, the predicted 45 OsMATE (*Oryza sativa* MATE protein) genes were located on 12 chromosomes. Chromosomes 3, 10, and 6 contained 9, 7, and 5 OsMATE genes, respectively, while chromosomes 2 and 5 had 1 OsMATE gene each. Chromosomes 4 and 11 contained 2 OsMATE genes each, chromosomes 7 and 9 contained 3 OsMATE genes each, and chromosomes 1, 8, and 12 contained 4 OsMATE genes each.

### Phylogenetic and structural analysis of MATE genes in rice and Arabidopsis

**Fig. 3:**
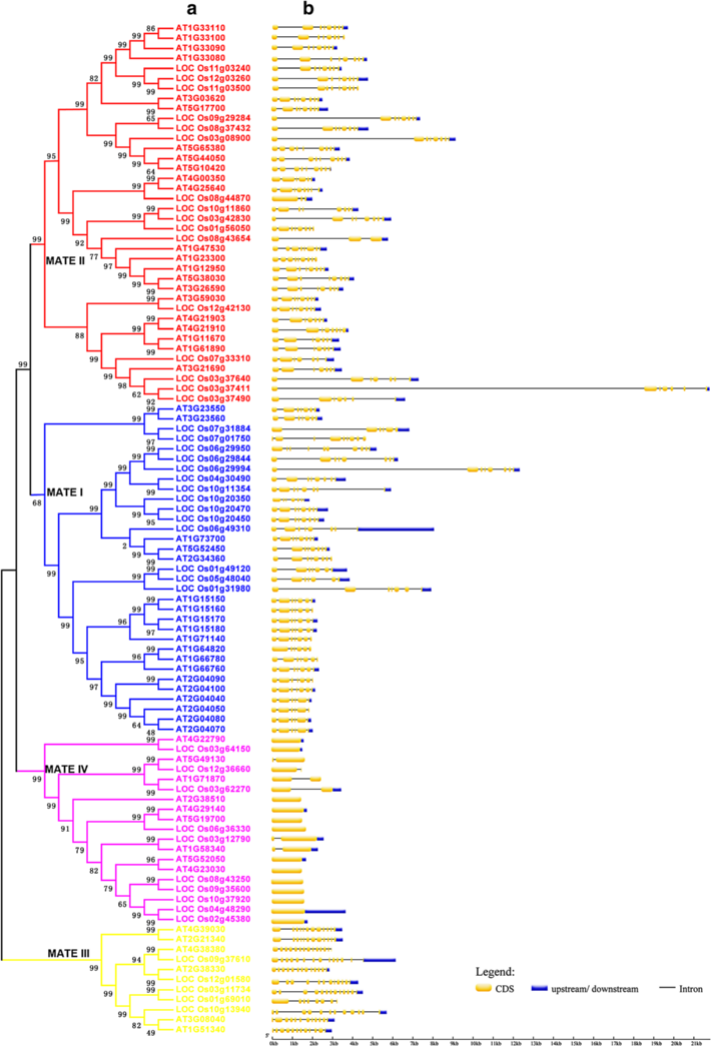
Phylogenetic relationships and exon-intron structure of MATE genes. **a** A neighbor-joining (N-J) phylogenetic tree was constructed using the complete protein sequence alignments of 101 MATE genes identified using MUSCLE and MEGA6. Numbers at the nodes represent bootstrap support values (1000 replicates). The color of the subclades indicates the four gene subfamilies. **b** Exon-intron structures of the MATE genes. Boxes, exons; lines, introns. The lengths of boxes and lines are scaled according to gene length

The program MUSCLE (Multiple Sequence Comparison by Log-Expectation) was employed to construct a multiple alignment of the identified 101 full-length protein sequences [27, 28]. The completed multiple alignment profiles of protein sequences were used to construct a phylogenetic tree with MEGA6.0 [29]. In addition, we employed three phylogenetic inference methods, namely neighbor-joining (N-J), minimum evolution (ME), and maximum likelihood (ML), to construct phylogenetic trees to confirm the topologies. All of these trees showed similar topologies; because the neighbor-joining (N-J) tree has higher bootstrap values than the other two phylogenetic trees. The N-J tree was employed for further analysis (Fig. 3). The topology of the N-J phylogenetic tree and the highest bootstrap values indicated that the MATE gene family could be divided into four major subfamilies: MATE I, MATE II, MATE III, and MATE IV. In order to explore the similarities and differences between members of the MATE gene family in rice and Arabidopsis, we constructed two N-J trees using the protein sequences of each species separately. Both trees had the same topology as that constructed using all 101 protein sequences (Additional files 7 and 8). All four MATE subgroups were present in both rice and Arabidopsis, indicating that these four subfamilies must have formed before the monocot-dicot split approximately 200 million years ago (Mya). The exon-intron organization of the MATE genes in the two species was examined by comparing the predicted coding sequences (CDSs) and their corresponding genomic sequences using GSDS software (http://gsds.cbi.pku.edu.cn/); this analysis was expected to provide more insight into the evolution of gene structures in the two species [30]. A majority of the genes of the MATE II subfamily (35 of 38; 92.1 %) had 6 to 8 introns (Fig. 3, Additional files 4 and 5). Similarly, 93.9 % (31 of 33) members of MATE I subfamily had 5 to 7 introns. However, all the genes in the MATE IV subfamily either lacked introns or had only a single intron; 13 genes had no introns and 6 genes had one intron. In contrast, 90.9 % (10 of 11) genes of MATE III subfamily had 11 to 13 introns: 5 genes had 11 introns, 2 genes had 12 introns, and 3 genes had 13 introns. Within the same subfamily, MATE genes of rice and Arabidopsis had similar intron numbers.

Based on previous research results and using the protein localization predictor WoLF PSORT [31], we obtained real or predicted subcellular location information for the MATE gene family in rice and Arabidopsis. As shown in Additional file 6, most protein members of MATE I and MATE II were predicted to be located in the plasma membrane, while some protein members of MATE III and MATE IV were predicted to be located in the chloroplast envelope membrane and plasma membrane, respectively [32–38]. In addition, a small number of MATE III and MATE IV protein members were predicted to be present in either the vacuolar membrane or cytoplasm. These results indicate that the proteins of different MATE subfamily members might have distinct subcellular locations.

To date, the functions of many MATE gene family members have been resolved in different plant species. In order to explore different MATE subfamilies members functional feature, we employed these MATE members and the identified 101 MATE members in this study to construct an N-J (neighbor-joining) phylogenetic tree. As shown in Additional files 9 and 10, we found that MATE gene members of the same subfamily have either the same or similar functions, while members of different subfamilies have disparate functions [14–16, 18–23, 26, 32–37, 39–62]. For example, in subfamily III, some MATE gene members gathered into one cluster (GmFRD3a [39] |GmFRD3b [39] |LjMATE1 [40] |At3g08040 [41, 42] |EcMATE1 [43] | HvAACT1 [63] |LOC_Os03g11734 [26] |LOC_Os01g69010 [32] | BoMATE [44] |At1g51340 [45] |ZmMATE1 [46] |VuMATE [47] |LOC_Os10g13940 [48] |SbMATE [23, 49, 50] |ScFRDL1 [51] |TaMATE1B [52]). All the MATE members of this cluster use citrate as a substrate and play an important role in plant aluminum tolerance and iron translocation. In contrast, the known funtional members of MATE subfamily II whose functions are known use flavonoids (proanthocyanidin, anthocyanin, or flavonoid) as substrates and are involved in transport of the corresponding substrates. Additional files 9 and 10 also demonstrate that MATE gene members of the same subfamilies have the same or similar substrate preferences and tissue and subcellular localizations; different subfamily members have different characteristics. This conclusion is consistent with previous reports. These results infer that functional divergence mainly took place between different MATE gene subfamily, which support the subsequent functional divergence analysis by the DIVERGE v3.0 program.

Overall, the analyses showed that the MATE gene family in rice and Arabidopsis showed consistent changes in intron patterns, and consequently, both species had similar exon-intron structures for genes in the same subfamily. In contrast, genes in different subfamilies showed dramatic divergence in exon-intron structures. The gene exon-intron structure characteristics in the two species also supported our classification results for MATE genes in rice and Arabidopsis.

### Duplication events in MATE gene family

It is well known that segmental duplication, tandem duplication, and retroposition are three important mechanisms of gene duplication [64]. However, although segmental duplication and tandem duplication have been shown to be important for the expansion of multigene families, the contribution of retroposition remains unclear [64].

Thus, in the present study, we focused on segmental and tandem duplications.

In rice, 15.6 % (7 of 45) of MATE family genes were considered to be derived from segmental duplication (Fig. 1); the corresponding value in Arabidopsis was 17.9 % (10 of 56) (Fig. 2). These results suggest that segmental duplication has made a similar contribution to the expansion of the MATE gene family in the two plant species.

Within the same or neighboring intergenetic regions, multiple members of one family could be generated through tandem duplication events. In the present study, adjacent homologous genes on a single chromosome, and with no more than 10 intervening genes between them, were defined as tandemly duplicated genes [65]. In rice, 20 % (9 of 45) MATE gene members were identified as tandem duplications (Fig. 1); in Arabidopsis, the corresponding value was 35.7 % (20 of 56) (Fig. 2). These results suggest that tandem duplication played an important role in the expansion of the MATE gene family in both rice and Arabidopsis.

To estimate the approximate ages of the segmental duplication events we used synonymous base substitution rates (*K*s values) as a proxy for time. As shown in Table 1, five pairs of segmental duplication genes were identified from rice and Arabidopsis. All five pairs of identified rice paralogous genes were predicted to have resulted from segmental duplication approximately 48–53.9 Mya, an estimate that is roughly consistent with the large-scale duplication events that occurred in the rice genome at approximately 40 Mya [66]. All five pairs of Arabidopsis MATE genes were estimated to have originated at 24.5–26.8 Mya; this estimate is roughly consistent with the occurrence of large-scale duplications at 28–48 Mya [67]. From the results of this analysis, we suggest that the segmentally duplicated genes in both rice and Arabidopsis were retained after the whole-genome duplication events that occurred during the evolution of both species. In addition, the two genes of each duplicated pair belonged to the same subfamily suggesting that they did not undergo evolutionary divergence after duplication.

**Table 1:** Estimates of the dates for the segmental duplication events of MATE gene family

Gene pairs		KS (mean ± s.d.)	Estimated time (mya)	GWD (mya)
AT2G21340	AT4G39030	0.735 ± 0.160	24.5	28-48
AT5G10420	AT5G65380	0.753 ± 0.160	25.1	
AT3G03620	AT5G17700	0.761 ± 0.143	25.4	
AT1G12950	AT3G26590	0.761 ± 0.157	25.4	
AT1G11670	AT1G61890	0.803 ± 0.129	26.8	
LOC_Os01g49120	LOC_Os05g48040	0.624 ± 0.163	48	30-40
LOC_Os02g45380	LOC_Os10g37920	0.678 ± 0.097	52.154	
LOC_Os02g45380	LOC_Os04g48290	0.701 ± 0.196	53.923	
LOC_Os04g48290	LOC_Os10g37920	0.633 ± 0.235	48.692	
LOC_Os08g37432	LOC_Os09g29284	0.625 ± 0.118	48.077	

We also submitted the sequences of the deduced tandem duplicated genes to the Plant Genome Duplication Database [68] to screen for tandem duplicated pairs in the two species. However, no homologous genes were found, which indicates that the tandem duplicated genes were retained after speciation of the two species studied.

Overall, both segmental duplication and tandem duplication events have made equally important contributions to the expansion of the MATE gene family in rice and Arabidopsis. In addition, the genes involved in segmental duplication in the two species appeared to have been retained after whole genome duplication in both species.

### Expression analysis of MATE genes in rice and Arabidopsis

**Fig. 4:**
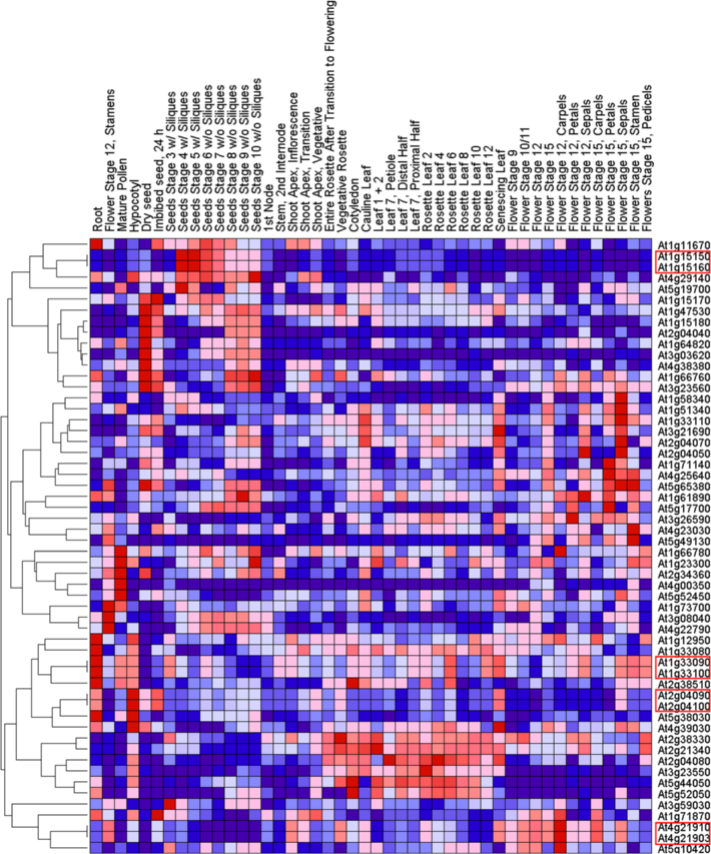
Expression profiles of *Arabidopsis thaliana* MATE genes. The level of expression is shown by the color and its intensity: deep red indicates the highest level of expression, deep blue the lowest. Other hues indicate intermediate levels of expression. The proteins highlighted by the red outlined boxes represent small phylogenetic clades that have similar transcript abundance profiles

**Fig. 5:**
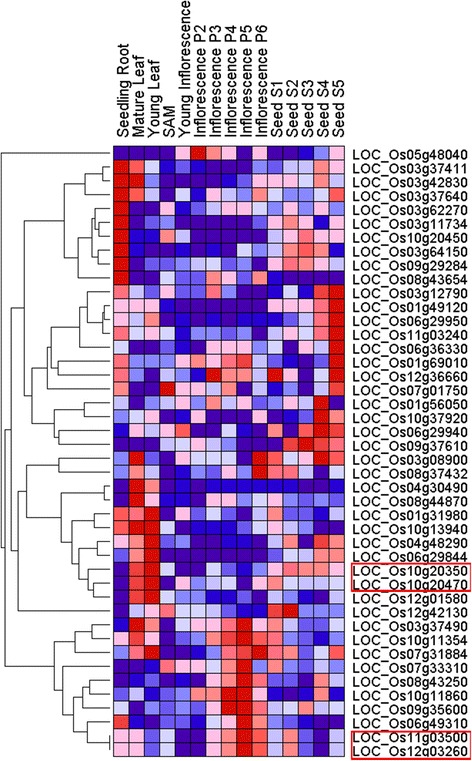
Expression profiles of rice MATE genes. The level of expression is shown by the color and its intensity: deep red indicates the highest level of expression, deep blue the lowest. Other hues indicate intermediate levels of expression. The proteins highlighted by the red outlined boxes represent small phylogenetic clades that have similar transcript abundance profiles

We compared the possible roles of homologous MATE genes in plant growth and development in rice and Arabidopsis by constructing heat maps using the Gene Pattern program [69]. The expression profiles indicated that most MATE family members of both species showed different expression levels in the tested tissues and organs (Figs. 4 and 5). Additionally, the MATE genes showed preferential expression: 84.4 % (38 of 45) and 85.7 % (48 of 56) of the MATE genes of rice and Arabidopsis, respectively, exhibited transcript abundance profiles with marked peaks in a single tissue. These results suggested that the MATE proteins function as tissue-specific regulators and are limited to discrete cells or organs. Approximately 17.8 %, 17.8 %, 20 %, and 26.7 % of MATE genes in rice showed their highest levels of transcript accumulation in the root, flower, leaf, and seed tissue, respectively. In Arabidopsis, approximately 8.9 %, 25 %, 12.5 %, and 25 % of MATE genes showed their highest levels of transcript accumulation in the root, flower, leaf and seed tissue, respectively. Surprisingly, only one rice MATE gene showed its highest level of transcript accumulation in the shoot apical meristem. In Arabidopsis, 3, 3, and 2 genes showed their highest levels of transcript accumulation in stamens, mature pollen, and the hypocotyl, respectively. The widely varying patterns of expression suggest that MATE genes in the two species are involved in the development of all tissues or organs under normal conditions. In addition, MATE genes that clustered in the branches of the heat map exhibited similar transcript abundance profiles. However, most MATE genes did not cluster in the phylogenetic tree but showed relatively distinct phylogenies. A few small phylogenetic clades had similar transcript abundance profiles; these are marked on the heat map by the red outlined boxes (Figs. 4 and 5). The genes in the two species that have high sequence similarity and share expression profiles represent good candidates for the evaluation of gene functions. We suggest that the genes in the red outlined boxes may have similar functions in the same tissues.

As shown in Additional file 12, approximately half of the AtMATE members are preferentially expressed in root tissues under stress conditions, while the remaining AtMATE members show preferential expression in shoot tissues. In contrast to Arabidopsis, some OsMATE members are expressed in both roots and shoots under drought or cold stress. In addition, some OsMATE members show lower levels of expression in roots and shoots under drought or cold stress (Additional file 11). These results demonstrate that the MATE gene family may play an important role in plant stress responses.

It is well known that gene duplication increases expression diversity and enables tissue or developmental specialization to evolve. Ohno’s classic model on the fate of duplicated genes [70] and the duplication degeneration complementation model (DDC) predict that one of the duplicates may gain a new function (neofunctionalization), lose its function (pseudogenization), or develop an overlapping redundant function and expression pattern (subfunctionalization) [71]. As shown in Fig. 5, one pair of duplicated genes, LOC_Os04g48290 and LOC_Os10g37920, exhibited the most redundant expression and developed opposite regulatory actions. LOC_Os04g48290 was expressed at high levels in the young leaf, but was expressed at a low level in seeds. In contrast, LOC_Os10g37920 was highly expressed in seeds, but was expressed at a very low level in the young leaf tissue. This effect indicates a case of subfunctionalization. Similar examples were found in the remaining duplicated genes. In addition, a pseudogenization process might have occurred in the duplicated Arabidopsis genes At5g10420 and At5g65380. The former showed noticeably weaker expression than the latter in the flower tissue. However, the fact that AT5g10420 still showed some expression in the flower tissue could indicate that pseudogenization was not complete. A similar phenomenon also occurred in the duplicated genes At1g12950 and At3g26590.

### Functional divergence in the MATE gene family

Type-I and Type-II functional divergence of clusters in the MATE family were estimated using the DIVERGE v3.0 program to determine whether amino acid substitutions in the MATE gene family have caused functional diversification [72–74]. The estimation was based on the neighbor-joining trees (Additional files 7 and 8), where four major protein subfamilies were clearly present and supported by highly significant bootstrap values.

First, we used a likelihood ratio test to identify whether a significant amount of Type-I functional divergence (θ_I_) had occurred between any of the specified pairs of MATE subfamily genes in rice or Arabidopsis. As shown in Table 2, the estimated likelihood ratio test (LRT) values of the six specified pairs of Arabidopsis MATE gene subfamilies ranged from 28.564 to 133.88; thus, we can reject the null hypothesis (no functional divergence; *P* < 0.01, d.f. = 1). Rather, the analysis provides statistical support for the hypothesis that there was a highly significant alteration to the selective constraints affecting the six pairs of Arabidopsis MATE gene subfamilies that resulted in subgroup-specific functional evolution after diversification. The rice gene pair OsMATE I/OsMATE II rejected the null hypothesis at *P* < 0.05; the LRT values of the remaining five pairs of rice MATE gene subfamilies ranged from 29.569 to 47.732 and rejected the null hypothesis (no functional divergence) at *P* < 0.01 (d.f. = 1).

**Table 2:** Functional divergence between subfamilies of the MATE gene family

Group1	Group2	Type-I			Type-II	
		θ_I_ ± s.e.	LRT	Qk > 0.9	θ_II_ ± s.e.	Qk > 0.9
AtMATE I	AtMATE II	0.271 ± 0.060	54.678**	8	−0.966 ± 0.428	0
AtMATE I	AtMATE III	0.485 ± 0.108	58.705**	8	−0.307 ± 0.490	0
AtMATE I	AtMATE IV	0.276 ± 0.067	28.564**	1	−0.771 ± 0.386	0
AtMATE II	AtMATE III	0.531 ± 0.100	133.880**	37	0.128 ± 0.353	160
AtMATE II	AtMATE IV	0.358 ± 0.063	94.623**	12	−0.278 ± 0.313	0
AtMATE III	AtMATE IV	0.363 ± 0.103	49.843**	7	0.318 ± 0.303	170
OsMATE I	OsMATE II	0.145 ± 0.062	9.317*	1	−1.205 ± 0.463	0
OsMATE I	OsMATE III	0.582 ± 0.121	47.732**	8	0.617 ± 0.170	223
OsMATE I	OsMATE IV	0.350 ± 0.078	35.562**	6	−0.105 ± 0.312	0
OsMATE II	OsMATE III	0.445 ± 0.112	45.642**	5	0.606 ± 0.174	202
OsMATE II	OsMATE IV	0.255 ± 0.068	29.569**	5	−0.364 ± 0.355	0
OsMATE III	OsMATE IV	0.556 ± 0.119	46.894**	8	0.778 ± 0.110	170

Note: θ_I_ and θ_II_, the coefficients of Type-I and Type-II functional divergence

LRT, Likelihood Ratio Statistic; for *P* < 0.05 was marked by *, *P* < 0.01 was marked by **

Qk, posterior probability

Next, we sought to determine whether Type-II functional divergence (θ_II_) had occurred among pairs of MATE subfamilies in rice and Arabidopsis. As shown in Table 2, Type-II (θ_II_) coefficients between subfamilies I/III, II/III, and III/IV were all significantly greater than zero in rice indicating that there were significant changes in amino acid properties between these subfamilies. The other three pairs of rice MATE subfamilies (I/II, I/IV, and II/IV) had coefficients less than zero. In Arabidopsis, however, with the exception of subfamilies IV/III, the Type-II coefficients between the pairs of MATE subfamilies did not differ significantly from zero, indicating no significant changes in amino acid properties between these subfamilies.

The posterior probability (Qk) of divergence was also determined for each amino acid site to identify those that are critical for functional divergence between MATE subfamilies in rice and Arabidopsis [75]. Residues with Qk < 0.9 were excluded to reduce false positives. As shown in Table 2 and Additional file 8, the number of critical amino acid sites (Qk > 0.9) for Type-I functional divergence ranged from 1 to 8 for rice MATE pairs. In comparison, the range was 1 to 32 in Arabidopsis (Table 2, Additional file 13).

Interestingly, 225, 202, and 170 critical amino acid sites for Type-II functional divergence (Qk > 0.99) were identified in the rice I/III, II/III, and IV/III MATE gene subfamily pairs (Table 2, Additional file 14). These results indicated that functional divergence between these groups in rice were mainly attributable to rapid changes in amino acid physiochemical properties and to a change in the evolutionary rate. For the other three rice MATE gene subfamily pairs (I/II, I/IV, and II/IV), no critical amino acid sites were identified (Table 2) suggesting that functional divergence between subgroup pairs I/II, I/IV, and II/IV could largely be attributed to a change in the evolutionary rate. In Arabidopsis, the II/III and IV/III Arabidopsis MATE gene subfamily pairs had 160 and 170 critical amino acid sites (Qk > 0.99). However, the II/III Arabidopsis MATE gene subfamily pair had a θ_II_ coefficient less than zero; this indicates that the identified Type-II related critical amino acid sites might be unreliable. We therefore suggest that the functional divergence between the III/IV pair can be attributed mainly to Type-II functional divergence and secondarily to Type-I functional divergence. Functional divergence in the Arabidopsis II/III pair can be mainly attributed to Type-I functional divergence. The remaining four Arabidopsis MATE gene subfamily pairs can also be attributed to Type-I functional divergence, as no Type-II related critical amino acid sites were identified and they all had θ_II_ coefficients less than zero.

In summary, in both rice and Arabidopsis, the MATE family shows consistent trends in functional divergence: highly significant Type-I functional divergence has occurred in each subfamily; however, Type-II functional divergence has also occurred between subfamily III and the other subfamilies. In addition, there were small differences between rice and Arabidopsis with respect to the extent of Type-II functional divergence. Type-II coefficients (θ_II_) between the three rice subfamily pairs I/III, II/III, and IV/III were all significantly greater than zero; however, only the Type-II coefficient for III/IV was significant in Arabidopsis. We therefore infer that functional divergence occurred mainly between MATE subfamily III and the other MATE subfamilies.

### Positive selection in MATE gene family

We applied site-specific likelihood models to the MATE gene family in rice and Arabidopsis (Additional file 15 and Additional file 16); these models assume variable selective pressure among sites but no variation among branches in the phylogeny [76–78]. We used two pairs of models, forming two LRTs: M0 (one-ratio) and M3 (discrete), and M7 (beta) and M8 (beta&ω). When the rice and Arabidopsis data sets were used in the analysis, the M3 metric was significantly better than the corresponding one-ratio model (*P* < 0.01, d.f. = 4), indicating that one category ω was insufficient to describe the variability in selection pressure across corresponding amino acid sites in rice and Arabidopsis gene families. The model M8 suggested 0.001 % of sites to be under positive selection with ω = 1.163 and 1.539, and identified 2 sites under positive selection in rice and 11 in Arabidopsis. However, the difference between M7 and M8 was not statistically significant in either species. This non-significance might be a consequence of a lack of power of the LRTs. It is worth noting that parameter estimates under model M8 (beta&ω), suggested the presence of sites under positive selection in both rice and Arabidopsis.

The “free-ratio” model assumes a different ω parameter for each branch in the tree, while the “one-ratio” model assumes the same ω ratio for all lineages. By comparing twice the log-likelihood difference between these two models, we can explore whether there are variable ω ratios among lineages in the rice and Arabidopsis MATE families. As shown in Tables 3 and 4, when these two models were applied to rice and Arabidopsis, all of the differences between the two models were significant, indicating that the ω ratios were extremely variable among lineages in both species. We performed a branch-site model analysis to test for positive selection affecting individual sites in different subfamilies of rice and Arabidopsis. On the MATE gene tree (Additional files 7 and 8), the four branches (I, II, III, and IV) were independently defined as the foreground branch in the two species. When each MATE subfamily was defined as the foreground branch in rice and Arabidopsis, the ratio ω_2_ was always significantly greater than one, suggesting that in both species each subgroup was under strong positive selection pressure (Tables 3 and 4). We also examined the posterior probability for site classes under model A to identify which sites were likely to be under positive selection in each subfamily. Critical positive selection sites were identified in OsMATE I, OsMATE II, OsMATE III, and OsMATE IV (Additional file 17). In Arabidopsis, the analysis identified critical positive selection sites in AtMATE I, AtMATE II, and AtMATE III but not in AtMATE IV (Additional file 18). In agreement with the foreground branch ratio ω_2_ results described above, we further suggest that there is significant positive selection (with ω > 1) acting at some sites in OsMATE I-IV and AtMATE I-III MATE subgroups.

**Table 3:** Parameters estimation and likelihood ratio tests for the branch-site and free-ratio models among Arabidopsis MATE genes

Cluster	Model	np^a^	lnL	2⊿l	Positive selected sites^b^
AtMATE I	Model A-null	114	−52052.69		Not allowed
	Model A	115	−52042.81	19.74**	9
AtMATE II	Model A-null	114	−52013.53		Not allowed
	Model A	115	−51982.21	62.64**	32
AtMATE III	Model A-null	114	−52053.21		Not allowed
	Model A	115	−52046.24	13.92**	6
AtMATE IV	Model A-null	114	−52053.82		Not allowed
	Model A	115	−52050.76	6.11*	none
Not allowed	M0: one-ratio	112	−52264.45		none
	Mf: free-ratio	221	−52043.51	441.86**	Not allowed

Note: *p* < 0.05 were marked by *, *p* < 0.01 were marked by **

^a^Number of parameters in the ω distribution

^b^The numbers of Positive-selection sites are inferred at posterior probabilities > 95 %

**Table 4:** Parameters estimation and likelihood ratio tests for the branch-site and free-ratio models among rice MATE genes

Cluster	Model	np^a^	lnL	2⊿l	Positive selected sites^b^
OsMATE I	Model A-null	92	−32162.29		Not allowed
	Model A	93	−32157.88	8.82**	2
OsMATE II	Model A-null	92	−32161.05		Not allowed
	Model A	93	−32158.60	4.90**	3
OsMATE III	Model A-null	92	−32155.71		Not allowed
	Model A	93	−32149.67	12.08**	115
OsMATE IV	Model A-null	92	−32161.59		Not allowed
	Model A	93	−32154.39	14.4**	7
Not allowed	M0: one-ratio	90	−32415.33		none
	Mf: free-ratio	177	−32148.55	533.56**	Not allowed

Note: *p* < 0.01 were marked by **

^a^Number of parameters in the ω distribution

^b^The numbers of Positive-selection sites are inferred at posterior probabilities > 95 %

Although each subfamily in rice and Arabidopsis was found by the branch-site model analysis to experience positive selection, the effects of selection were different in each subfamily of rice and Arabidopsis; thus, the MATE II subfamily in Arabidopsis experienced higher positive selection than the other three Arabidopsis subgroups, while the MATE III subgroup in rice had greater positive selection than the other subfamilies. In addition, considering the LRTs and the Bayes empirical Bayes (BEB) calculations of posterior probabilities, we suggest that positive selection has affected the evolution of each MATE subfamily in both rice and Arabidopsis.

### Coevolution of MATE amino acid sites

Protein evolution depends on an intramolecular co-evolutionary network, the complexity of which is proportional to the underlying functional and structural interactions among sites [79]. The more complex the co-evolutionary network of a particular site, the greater the selection coefficient can be against a mutation at that site due to the dramatic effect the mutation might have on other coevolving protein regions. Testing for coevolution between sites is thus an essential step to complement molecular selection analysis and provide more biologically realistic results.

**Fig. 6:**
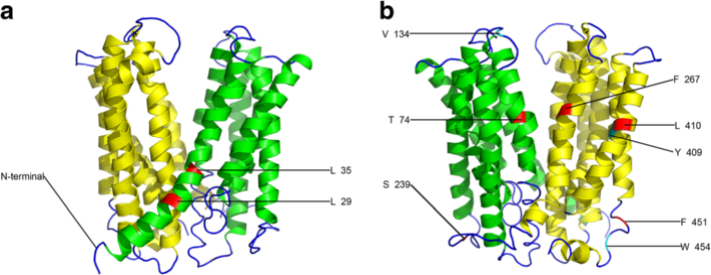
Model building of the 3D structure of Arabidopsis MATE protein AT1G73700. **a**, **b** Seven critical amino acid sites responsible for both functional divergence and positive selection and three sites responsible for coevolution are shown to a varying degree. The figure was produced using the Phyre2 and pyMOL programs. Sites responsible for both functional divergence and positive selection are colored red, while three responsible for coevolution are colored cyan

**Fig. 7:**
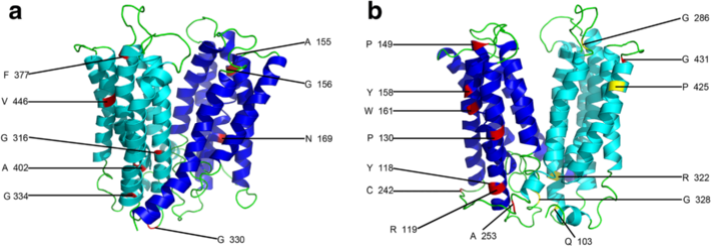
Model building of the 3D structure of rice MATE protein LOC_Os01g49120. **a**, **b** Eighteen critical amino acid sites responsible for both functional divergence and positive selection and 5 sites responsible for coevolution are shown to a varying degree. The figure was produced using the Phyre2 and pyMOL programs. The amino acid sites responsible for both functional divergence and positive selection are colored red, while those responsible for coevolution are colored yellow

We identified a group of amino acids showing coevolution in rice and another in Arabidopsis (Additional file 19). As shown in Fig. 6, there are three critical sites in the Arabidopsis MATE coevolving amino acids: 34 V, 409Y, and 454 W. These sites are far apart, suggesting functional dependency between them. Interestingly, in the five critical sites in rice with coevolving amino acids (Fig. 7), three sites (103Q, 322R, and 328G) were highly proximal and the other sites (286G and 425P) were also spatially proximal; thus, compensatory mutations might maintain the stability of the local structure, which might also be an indicator of functional coevolution.

### Identification of critical amino acid sites

**Fig. 8:**
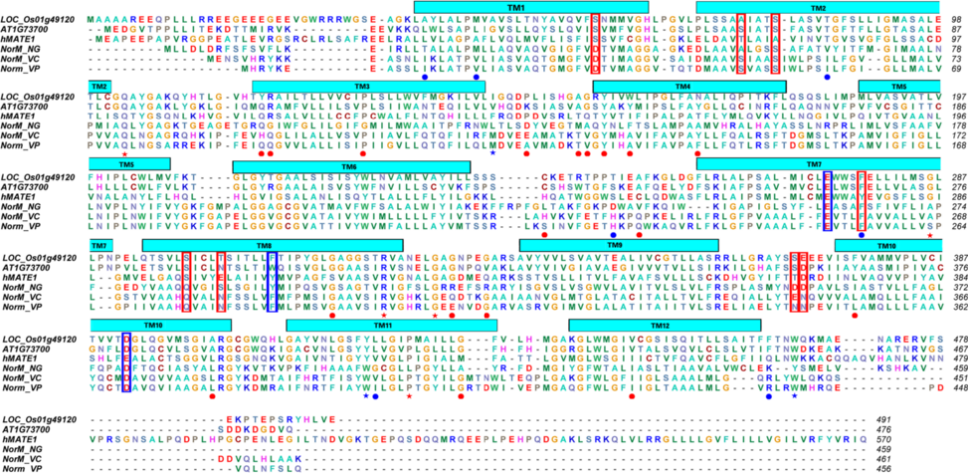
Multiple sequence alignment of several MATE protein sequences. Sequence alignment of the reference proteins (LOC_Os01g49120 and AT1G73700) with NorM-NG, NorM-VC, NorM-VP, and hMATE1. Regions of transmembrane helices (TM) in LOC_Os01g49120 are outlined and numbered. Blue dots and triangles indicate amino acids responsible for functional divergence, positive selection and coevolution in the reference sequence AT1G73700. Red dots and triangles indicate amino acids responsible for functional divergence, positive selection, and coevolution in the reference sequence LOC_Os01g49120. The cyan and red outlined boxes represent amino acids that might coordinate cations and interact with substrates, respectively

We identified 18 and 7 critical sites in rice and Arabidopsis, respectively, that were responsible for positive selection, and Type-I, and Type-II functional divergence (Figs. 6 and 7). These sites were located on the corresponding three-dimensional OsMATE and AtMATE structures. We performed a multiple sequence alignment to further investigate rice and Arabidopsis MATE protein functions (Fig. 8). Among the 18 identified rice critical sites, TM3 (transmembrane helices 3), L3-4 (linker between TM3 and TM4), and TM4 were observed at three sites each; L6-L7, L8-L9, and TM10 each had two critical sites; and TM8, TM11, and TM12 each had one critical site (Figs. 7 and 8). Among the 7 Arabidopsis critical sites, TM1 had two sites, and TM2, L6-7, TM7, TM11 each had one site; the C-terminus also had one site. The results suggested that these critical amino acids might make an important contribution to the adaptiveness of MATE proteins for different functional needs in rice and Arabidopsis.

Additionally, one critical coevolving site was present in each of L3-4, TM11, and the C-terminus in rice. In Arabidopsis, L2-3, T7, T8, L8-9, and T11 had one critical coevolving site each.

These analyses indicate that the numbers and locations of the identified critical amino acid sites in rice were different from those of Arabidopsis. Thus, functional divergence, positive selection, and coevolution might act in different regions of rice and Arabidopsis MATE gene proteins.

## Discussion

### Comparative genomic analysis of the MATE gene family in rice and Arabidopsis

In this study, we identified 45 and 56 MATE genes from rice and Arabidopsis, respectively. Phylogenetic tree analysis (Fig. 3) showed that these MATE genes belonged to four major subfamilies, groups I–IV. We examined intron evolution in these genes as this is an important feature of genomic evolution as well as being an adaptive process in speciation. Interestingly, the results indicated that in the same subfamily, gene members exhibited similar exon-intron structures and similar numbers of introns, while in different subgroups, gene members displayed significantly different exon-intron structures. The loss or gain of introns over a long evolutionary period might be the critical reason underlying gene structure variation. Additionally, these results supported our classification of MATE gene subfamilies in rice and Arabidopsis. We also conclude that since all four MATE subfamilies are present in rice and Arabidopsis then they must have formed before the monocot-dicot split approximately 200 Mya.

Tandem duplication is likely an important process in adaptive evolution under rapidly changing environments, although genes involved in stress responses may have a high probability of retention following tandem duplication [80]. A number of studies have reported that transporter proteins from the MATE family play vital roles in metabolite transport in plants, and have a critical influence on yield in crop species [23, 53]. MATE transporters also mediate multidrug resistance in bacteria and animals [81] and modulate the efficacy of many pharmaceutical drugs used in the treatment of various diseases [82–85]. Our study revealed the involvement of tandem duplication events in 20 % (9 of 45) of rice MATE genes and 35.7 % (20 of 56) of Arabidopsis MATE genes, supporting the findings of Hanada et al. [80].

The retention of segmentally duplicated genes in rice and Arabidopsis after whole genome duplication indicates that large-scale duplication may also have been involved in the expansion of the MATE gene family in both species. In addition, MATE genes from these two species shared a common expansion model. Both tandem and segmental duplication played similar important roles in rice and Arabidopsis. Intriguingly, pairs of genes derived from tandem duplication or segmental duplication events belonged to the same subfamilies, suggesting that they had not undergone evolutionary divergence after duplication. The estimated dates of origin of all deduced MATE paralogous gene pairs ranged from 53.9 to 24.5 Mya (Table 1), and all deduced tandem duplicated genes may have originated after the formation of rice and Arabidopsis. Taken together, our results clearly indicate that these MATE duplicated genes postdate the split between monocots and dicots, which is thought to have occurred approximately 200 Mya.

### Expression analysis of MATE proteins

Most MATE family members in rice and Arabidopsis show variable expression in different tissues and organs (Figs. 4 and 5). These differences in the species suggested that different MATE genes might play specific roles in the development of tissues and organs under normal conditions. For example, LOC_Os3g11734 is mainly expressed in the roots and is necessary for efficient translocation of Fe to the shoot via Fe-citrate complexes [26]. The level of LOC_Os1g69010 expression is very low in different tissues under normal conditions [32]. However, in the heatmap produced here, the relative level of expression of this gene varied among tissues. Although relatively high levels of expression were found in seeds and roots compared to other tissues, the absolute level of expression was low. The level of LOC_Os1g69010 expression in roots is very low in the absence of Al, but increases considerably after short exposure to Al [32]. This report is consistent with our study that this gene has low expression levels in all tested tissues. A previous study showed that the MATE gene AT3G59030 is involved in the flavonoid biosynthetic pathway and is expressed specifically in ovules and developing seeds [16], which corroborate our results that the highest level of transcription of AT3G59030 occurred in seeds.

Yamasaki et al. [54] reported that AT4G39030 is specifically localized to the chloroplast envelope membrane in Arabidopsis and is responsible for transport of salicylic acid from the chloroplast to the cytoplasm in epidermal cells. Here, we found that this gene was preferentially expressed in hypocotyls and in senescing leaf tissues. Salicylic acid is a phytohormone that plays a critical role in plant immunity, indicating that AT4G39030 might have an important role in the hypocotyl and in senescing leaf tissue. Expression of the AT3G23560 gene is required for the protection of roots against inhibitory compounds [15]. Transcriptional and translational fusion of the gene to a B-glucuronidase reporter gene showed that it is expressed strongly in the root epidermis, a tissue in direct contact with the external environment. Here, however, our profile of transcript abundance profile showed that AT3G23560 peaked in dry seeds with a lower level of expression in root tissue. Thus, further investigation is needed to determine whether this MATE protein has an important role in dry seeds.

As the expression profiles of MATE genes in rice and Arabidopsis differ among tissues and organs, then it is likely that the functional regions of the MATE genes have also diverged. Significantly, our results also demonstrated expression divergence in MATE duplicated genes during the evolution of the two species.

### Functional divergence, positive selection, and coevolution analysis

Unfortunately, the predicted 3-D structures of MATE proteins with known functions were not appropriate. As a consequence, we choose to compare LOC_Os01g49120 and At1g73700 as these had high-quality predicted protein 3-D structures. We identified 18 and 7 critical amino acids in rice and Arabidopsis, respectively, when we analyzed the relationship between positive selection and functional divergence. These critical sites were positioned on three-dimensional MATE structures (Figs. 6 and 7) and we performed multiple sequence alignment to further investigate their functions (Fig. 8). The protein sequences NorM_NG, NorM_VC, and NorM_VP have been studied previously with regard to structure and function [86–89]; we therefore used these protein sequences in multiple sequence alignments with two MATE reference sequences to investigate the MATE family in the two species. As shown in Fig. 8, three amino acids were found to coordinate cations and eight amino acids were shown to interact with substrates. Lu et al. [88] showed that the outward-facing, drug-bound NorM-NG uses E261 and Y294 (equivalent to LOC_Os01g49120^E274 and F309^ and AT1G73700^E263 and W298^) to initiate Na + loading from the extracellular side. Subsequently, D377 (equivalent to LOC_Os01g49120^D392^ and AT1G73700^D381^) participates in Na + coordination as TM7 and TM8 approach TM10. NorM_VP^E251 and D367^ and NorM _VC^E255 and D371^ have been suggested to be Na + −coordinating residues, and these sites are the counterparts of NorM-NG^E261 and D377^ [86, 87, 89]. Mutated hMATE1, which has the amino acid replacement E273Q, is the counterpart of E261 found in the bacterial NorM_NG protein [86]. All these results suggest that the residues of LOC_Os01g49120^E274, F309, and D392^ and AT1G73700^E263, W298, D381^ are crucial for the recognition and binding of cations. When mapping these sites on the corresponding 3-D MATE structures (Additional files 20 and 21), we found that these amino acids side chains extended into each other, implying that these critical amino acids were responsible for cation coordination. Additionally, all eight amino acids that are expected to interact with substrates were located around the suggested central substrate-binding cavity indicating the importance of these amino acids in this aspect of substrate transportation. We inferred that AT1G73700^F267^ interacted with substrates [86–89]. Interestingly, we also found that the AT1G73700^F267^ amino acid site experienced both functional divergence and positive selection. This is one of the reasons why MATE members have a broad ability to extrude structurally and chemically distinct drugs from cells.

Coevolution analysis of MATE proteins identified a group of affected amino acids in both species (Additional file 19). Interestingly, in the LOC_Os01g49120 coevolving sites (Fig. 7), 103Q, 322R, and 328G were all spatially proximal around the base of the central substrate-bounding cavity, although they are distant on the primary structure. This suggests that compensatory mutations in these sites have probably maintained the highly ordered protein structural stability of the cavity bottom where the cavity is shielded from the cytoplasm. However, these three critical coevolving amino acids were spatially distant to 286G and 425P, suggesting a functional dependency between these five coevolving amino acids in LOC_Os01g49120. In a similar manner to this rice protein, the three coevolving amino acids of AT1G73700, which were spatially proximal to each other, might also contribute to the dependency between important functional domains. Some critical amino acid sites, identified from positive selection and functional divergence analyses, were located around the coevolving amino acids in MATE proteins. This observation further underlines the importance of these amino acids for realizing MATE protein biological functions.

### Possible origins of MATE subfamilies in plants

As for MATE III subfamily, although some researchers took this subfamily into two or three groups, we thought it is more reasonable to be in one subfamily. Firstly, gene structure analysis showed that they shared almost the same intron-exon structure (10 of 11 members of this subfamily have 11–13 introns), which is distinctly different from the other subfamily. Secondly, the highest bootstrap values of phylogenetic tree suggested that MATE III subfamily members have extremely high sequence similarity. Thirdly, in addition to At4g39030, which used salicylic acid as substrate, all the other known function subfamily III members used citrate as substrate. Thus, we believe that we take the members of MATE III as one whole subfamily is reasonable. In addition, according to the Additional file 6, we can know that some gene members in subfamily III have different subcellular localization indicating functional divergence have taken place in this subfamily. All the above results demonstrate the gene structures of MATE III subfamily members were conservative, but functional divergence might led to this subfamily members functional diversification.

Previous phylogenetic analysis divided the MATE-type transporter family into three subfamilies, bacterial (family 1), eukaryotic (family 2), and bacterial and archaea (family 3) [12, 90]. Eukaryotic family 2 was further divided into four subgroups: yeast and fungi, plant, animal and protozoan MATE subgroups. Interestingly, the members of MATE subfamily I, II, and IV in this study exactly belong to the plant-specific subgroup of family 2 (family 2 plants MATE), while subfamily III members belong to the bacteria/archaea family 3 (family 3 plants MATE) [12, 90]. These results might be explained with the assumption proposed by Yamasaki et al. [54], which conjectured that family 3 plants MATE transporters might be derived from bacterial proteins probably through endosymbiotic gene transfer. Thus, we speculated that the MATE III subfamily might have a different origin from the other MATE subfamilies in our study. Our functional divergence analysis showed that Type-II divergence was exclusive to the MATE III subfamily in both rice and Arabidopsis, indicating that site-specific amino acid physiochemical properties in this subfamily have diverged from those of the other subfamilies. As shown in Additional files 8 and 9, a number of Type-II functional divergence critical amino acids were identified in our analysis, although we did not identify any Type-II functional divergence sites in subfamilies I, II, and IV in the two species. Thus, our results further demonstrate that the MATE III subfamily in plants might have a different origin from the other three MATE subfamilies.

## Conclusions

In this study, we identified 45 and 56 MATE genes in the model monocot *Oryza sativa*, and the model dicot *Arabidopsis thaliana*, respectively. The results of our analyses indicate that both tandem and segmental duplications have contributed to the expansion of the MATE gene family in these species and that similar expansion processes occurred in both species. All of the putative duplicated genes in these two species postdate the monocot-dicot split. Furthermore, differential expression of duplicated MATE genes in rice and Arabidopsis suggested that protein functions might have diverged to meet special requirements. The branch-site model showed that the members of each subfamily experienced high positive selection pressure in both species, leading to subfamily-specific functional evolution. Analysis of Type-II functional divergence showed that the critical amino acids were always identified when subfamily III was compared with other groups, strongly suggesting that changes in site-specific amino acid physiochemical properties might be attributable to subfamily III-specific functional evolution in both species. Co-evolutionary analysis indicated that coevolving sites might have an important role in maintaining local structural stability and function of protein functional domains in the rice and Arabidopsis MATE gene family. The results of this study contribute to an improved understanding of the complexity of the MATE gene family and provide insights into the functional and evolutionary similarities and dissimilarities between these two model plant species.

## Methods

### Identification of MATE genes

Fifty-six *Arabidopsis thaliana* MATE protein sequences were downloaded from the Phytozome database (https://phytozome.jgi.doe.gov/pz/portal.html) and were used in a BLAST search against *Oryza sativa* sequences (http://rapdb.dna.affrc.go.jp/), using the BLASTP program. Sequences were selected as candidate proteins if their E value was ≤1e-5. The Simple Modular Architecture Research Tool (SMART; http://smart.embl-heidelberg.de/smart/batch.pl) and Pfam (http://pfam.xfam.org/) were used to confirm each predicted MATE protein sequence. Redundant and partial genes were manually removed. For each query sequence, information on genomic sequences, full coding sequences, and protein sequences were collected from the corresponding databases.

### Alignment, phylogenetic analysis, exon-intron structure motif analysis and promoter analysis

The identified MATE proteins were aligned using the MUSCLE program with the default parameters. The neighbor-joining method and MEGA6.0 were used to infer phylogenetic trees and the reliability of interior branches was assessed with 1000-bootstrap samples. The online Gene Structure Display Server (GSDS: http://gsds.cbi.pku.edu.cn/) was employed to explore the diagrams of exon-intron structure with the coding sequence (CDS) and genomic sequence.

### Dating duplication events

Ka and Ks for corresponding duplicated gene pairs were obtained directly from The Plant Genome Duplication Database (http://chibba.agtec.uga.edu/duplication/) [68]. Detailed information on the anchor points of duplicated gene pairs and the mean Ks values have been described previously [91, 92]. The approximate date of the duplication event was calculated using the mean Ks values from T = Ks/2λ [93], assuming clocklike rates (λ) of synonymous substitution of 6.5 × 10^−9^ for rice [66] and 1.5 × 10^−8^ for Arabidopsis [94].

### Tests of positive selection

Positive selection was identified using a maximum likelihood approach by “codeml” in PAML under the site model and branch-site model [76–78]. The two model analysis of MATE family and subfamilies was performed according to the previously described method [92].

### Estimation of functional divergence

Functional divergence and the importance of amino acid residues among MATE gene subfamilies were predicted using the DIVERGE v3.0 package [72–74, 95, 96], which estimates significant changes in site-specific shifts according to evolutionary rate (Type-I) or amino acid properties (Type-II) after the emergence of two paralogous sequences. Detailed information on the estimation of functional divergence has been described previously [92].

### Analysis of MATE coevolution

Coevolution analysis using protein sequences (CAPS) was performed to identify coevolution between amino acid sites [79]. Detailed information on the coevolution analysis has been described previously [97].

### Extraction of microarray data

The rice eFP Browser (http://www.bar.utoronto.ca/efprice/cgi-bin/efpWeb.cgi) tool was used to search microarray data from rice. We also used data from experiment GSE6893 (Rice Genome Annotation Project) that analyzed spatial and temporal gene expression in various tissues and various stages of reproductive development of rice [98]. The expression values from the following tissues and development stages were retrieved: young leaf, mature leaf, stem apical meristem, panicle stages P1 to P6, seedling root, and seed stages S1 to S5. Information on expression of Arabidopsis MATE genes under various stress conditions were also obtained from experiment GSE6893 [98]. The data were normalized by MAS.5.0 and the RMA method. A target intensity value of 100 was used, and all tissues were sampled in triplicate.

The microarray data for Arabidopsis were obtained from the Arabidopsis eFP Browser (http://bar.utoronto.ca/efp/cgi-bin/efpWeb.cgi) tool. The data source was Development Map [99]. Expression values were retrieved and data were normalized by the GCOS method, with a target intensity value of 100. The tissues were sampled in triplicate. Information on expression of Arabidopsis MATE genes under various stress conditions were obtained from AtGenExpress Abiotic Stress Series [100]. Heat maps were generated using the Gene pattern program (http://www.broadinstitute.org/cancer/software/genepattern/).

## Additional files

Additional file 1: Protein sequence data of the MATE gene family. (TXT 52 kb)
Additional file 2: Coding sequence data of the MATE gene family. (TXT 154 kb)
Additional file 3: Genome sequence data of the MATE gene family. (TXT 364 kb)
Additional file 4: Predicted rice genes and related information. a. aa = amino acids; b. pI = isoelectric point of the deduced polypeptide; c. Mw = molecular weight; d. number of introns. (DOC 87 kb)
Additional file 5: Predicted Arabidopsis genes and related information. a. aa = amino acids; b. pI = isoelectric point of the deduced polypeptide; c. Mw = molecular weight; d. number of introns. (DOC 96 kb)
Additional file 6: Prediction of MATE protein members subcellular locations. (DOC 111 kb)
Additional file 7: The N-J phylogenetic tree of Arabidopsis MATE gene family. The neighbor-joining (N-J) phylogenetic tree was constructed based on a complete protein sequence alignment of 56 *Arabidopsis thaliana* MATE genes identified using MUSCLE and MEGA6. Numbers at the nodes represent bootstrap support values (1000 replicates). The color of subclades indicates the four corresponding gene subfamilies. (TIF 297 kb)
Additional file 8: The N-J phylogenetic tree of rice MATE gene family. The neighbor-joining (N-J) phylogenetic tree was constructed based on a complete protein sequence alignment of 45 rice MATE genes identified using MUSCLE and MEGA6. Numbers at the nodes represent bootstrap support values (1000 replicates). The color of subclades indicates the four corresponding gene subfamilies. (TIF 342 kb)
Additional file 9: The N-J phylogenetic tree of plant functionally known MATE menbers and 101 identified members from this study. (TIF 6275 kb)
Additional file 10: The information about plant functionally known MATE members. (DOC 98 kb)
Additional file 11: Expression profiles of rice MATE genes under various stress. (TIFF 658 kb)
Additional file 12: Expression profiles of Arabidopsis MATE genes under various stress. (TIFF 5235 kb)
Additional file 13: Type-I functional divergence sites identified between different subgroup pairs of rice and Arabidopsis. (DOC 20 kb)
Additional file 14: Type-II functional divergence sites identified between different subgroup pairs of rice and Arabidopsis. (DOC 30 kb)
Additional file 15: Tests for positive selection among codons of Arabidopsis MATE genes using site models. (DOC 19 kb)
Additional file 16: Tests for positive selection among codons of rice MATE genes using site models. (DOC 19 kb)
Additional file 17: Critical sites identified from different rice MATE subgroups by the branch-site model. Note: ^a^Positive-selection sites are inferred at posterior probabilities >95 % with those reaching 99 % shown in bold. (DOC 18 kb)
Additional file 18: Critical sites identified from different Arabidopsis MATE subgroups by the branch-site model. Note: ^a^Positive-selection sites are inferred at posterior probabilities >95 % with those reaching 99 % shown in bold. (DOC 15 kb)
Additional file 19: Critical coevolving amino acids identified in rice and *Arabidopsis thaliana*. (DOC 11 kb)
Additional file 20: The 3D structure of rice MATE protein LOC_Os01g49120. Amino acid sites that might interact with substrates are colored red, while those that might coordinate cation movements are colored yellow. (TIF 3471 kb)
Additional file 21: The 3D structure of *Arabidopsis thaliana* MATE protein AT1G73700. Amino acid sites that might interact with substrates are colored red, while those that might coordinate cation movement are colored cyan. (TIF 3069 kb)

## Abbreviations

ABC: ATP-binding cassette
AtMATE: Arabidopsis thaliana MATE protein
BEB: Bayes empirical Bayes
CAPS: Coevolution analysis using protein sequence
CDS: Coding sequence
DDC: Duplication degeneration complementation model
GSDS: Gene Structure Display Server
Ks values: Synonymous base substitution rates
LRT: Likelihood ratio test
MATE: Multidrug and toxic compound extrusion
ME: Minimum evolution
MFS: Major facilitator superfamily
ML: Maximum likelihood
MUSCLE: Multiple Sequence Comparison by Log-Expectation
Mya: Million years ago
N-J: Neighbor-joining
OsMATE: Oryza sativa MATE protein
PGDD: Plant Genome Duplication Database
Qk: Posterior probability
RND: Resistance-nodulation-division
SMART: Simple Modular Architecture Research Tool
SMR: Small multidrug resistance transporters
TEA: Tetraethylammonium
TM: Transmembrane helices
