# Supplementary material for: The similar and different evolutionary trends of MATE family occurred between rice and Arabidopsis thaliana
Source: BMC Plant Biol. 2016 Sep 26;16:207. doi: 10.1186/s12870-016-0895-0 (PMC5037600; doi:10.1186/s12870-016-0895-0)
Supplement: Additional file 4: — Predicted rice genes and related information. a. aa = amino acids; b. pI = isoelectric point of the deduced polypeptide; c. Mw = molecular weight; d. number of introns. (DOC 87 kb) [file 12870_2016_895_MOESM4_ESM.doc]

**Additional file 4. Predicted rice genes and related information**

| Group | Gene ID | Chromosome | ORF(aa)^a^ | pI^b^ | Mw(KD)^c^ | Introns^d^ |
| --- | --- | --- | --- | --- | --- | --- |
| I | LOC_Os01g31980 | 1 | 502 | 5.41 | 53.6 | 6 |
| I | LOC_Os01g49120 | 1 | 491 | 7.15 | 53.0 | 6 |
| I | LOC_Os04g30490 | 4 | 483 | 7.58 | 51.0 | 7 |
| I | LOC_Os05g48040 | 5 | 500 | 7.38 | 53.5 | 6 |
| I | LOC_Os06g29844 | 6 | 490 | 6.50 | 52.1 | 7 |
| I | LOC_Os06g29950 | 6 | 495 | 8.69 | 54.1 | 9 |
| I | LOC_Os06g29994 | 6 | 479 | 7.93 | 51.1 | 7 |
| I | LOC_Os06g49310 | 6 | 483 | 6.74 | 52.0 | 7 |
| I | LOC_Os07g01750 | 7 | 568 | 9.89 | 62.4 | 8 |
| I | LOC_Os07g31884 | 7 | 566 | 9.38 | 61.1 | 5 |
| I | LOC_Os10g11354 | 10 | 485 | 7.83 | 51.5 | 7 |
| I | LOC_Os10g20350 | 10 | 392 | 8.15 | 41.3 | 6 |
| I | LOC_Os10g20450 | 10 | 477 | 8.55 | 50.7 | 7 |
| I | LOC_Os10g20470 | 10 | 486 | 8.31 | 51.4 | 7 |
| II | LOC_Os01g56050 | 1 | 484 | 9.87 | 51.6 | 7 |
| II | LOC_Os03g08900 | 3 | 489 | 7.31 | 53.0 | 7 |
| II | LOC_Os03g37411 | 3 | 500 | 7.35 | 53.8 | 6 |
| II | LOC_Os03g37490 | 3 | 520 | 5.01 | 55.8 | 6 |
| II | LOC_Os03g37640 | 3 | 500 | 6.96 | 53.6 | 6 |
| II | LOC_Os03g42830 | 3 | 477 | 8.92 | 51.8 | 7 |
| II | LOC_Os07g33310 | 7 | 493 | 8.46 | 51.9 | 6 |
| II | LOC_Os08g37432 | 8 | 489 | 6.51 | 52.6 | 7 |
| II | LOC_Os08g43654 | 8 | 522 | 7.20 | 56.2 | 2 |
| II | LOC_Os08g44870 | 8 | 489 | 5.84 | 51.6 | 2 |
| II | LOC_Os09g29284 | 8 | 482 | 8.97 | 52.0 | 7 |
| II | LOC_Os10g11860 | 10 | 464 | 8.73 | 50.2 | 7 |
| II | LOC_Os11g03500 | 11 | 495 | 6.77 | 54.2 | 8 |
| II | LOC_Os11g03240 | 11 | 497 | 6.75 | 54.7 | 7 |
| II | LOC_Os12g03260 | 12 | 507 | 6.40 | 55.4 | 7 |
| II | LOC_Os12g42130 | 12 | 500 | 6.75 | 54.1 | 7 |
| III | LOC_Os01g69010 | 1 | 566 | 9.44 | 58.3 | 7 |
| III | LOC_Os03g11734 | 3 | 571 | 8.36 | 60.0 | 12 |
| III | LOC_Os09g37610 | 9 | 577 | 9.64 | 60.1 | 13 |
| III | LOC_Os10g13940 | 10 | 537 | 6.92 | 57.7 | 11 |
| III | LOC_Os12g01580 | 12 | 550 | 5.14 | 58.4 | 12 |
| IV | LOC_Os02g45380 | 2 | 549 | 7.35 | 57.8 | 0 |
| IV | LOC_Os03g12790 | 3 | 644 | 6.95 | 65.8 | 1 |
| IV | LOC_Os03g62270 | 3 | 516 | 7.28 | 53.5 | 1 |
| IV | LOC_Os03g64150 | 3 | 479 | 10.07 | 50.6 | 0 |
| IV | LOC_Os04g48290 | 4 | 560 | 6.74 | 59.2 | 0 |
| IV | LOC_Os06g36330 | 6 | 568 | 7.19 | 58.9 | 0 |
| IV | LOC_Os08g43250 | 8 | 536 | 8.35 | 55.7 | 0 |
| IV | LOC_Os09g35600 | 9 | 541 | 7.55 | 56.4 | 0 |
| IV | LOC_Os10g37920 | 10 | 539 | 7.35 | 56.0 | 0 |
| IV | LOC_Os12g36660 | 12 | 431 | 8.94 | 45.3 | 1 |
