# Supplementary material for: The similar and different evolutionary trends of MATE family occurred between rice and Arabidopsis thaliana
Source: BMC Plant Biol. 2016 Sep 26;16:207. doi: 10.1186/s12870-016-0895-0 (PMC5037600; doi:10.1186/s12870-016-0895-0)
Supplement: Additional file 5: — Predicted Arabidopsis genes and related information. a. aa = amino acids; b. pI = isoelectric point of the deduced polypeptide; c. Mw = molecular weight; d. number of introns. (DOC 96 kb) [file 12870_2016_895_MOESM5_ESM.doc]

**Additional file 5. Predicted Arabidopsis genes and related information**

| Group | Gene ID | Chromosome | ORF(aa)^a^ | pI^b^ | Mw(KD)^c^ | Introns^d^ |
| --- | --- | --- | --- | --- | --- | --- |
| I | AT1G15150 | 1 | 487 | 7.21 | 53.0 | 6 |
| I | AT1G15160 | 1 | 487 | 6.69 | 52.8 | 6 |
| I | AT1G15170 | 1 | 481 | 6.60 | 52.2 | 6 |
| I | AT1G15180 | 1 | 482 | 6.81 | 51.8 | 6 |
| I | AT1G64820 | 1 | 502 | 7.15 | 55.7 | 5 |
| I | AT1G66760 | 1 | 482 | 8.14 | 53.1 | 6 |
| I | AT1G66780 | 1 | 485 | 5.97 | 53.6 | 6 |
| I | AT1G71140 | 1 | 485 | 6.36 | 52.4 | 6 |
| I | AT1G73700 | 1 | 476 | 8.07 | 51.1 | 7 |
| I | AT2G04040 | 2 | 476 | 7.74 | 51.8 | 5 |
| I | AT2G04050 | 2 | 476 | 7.89 | 51.5 | 5 |
| I | AT2G04070 | 2 | 476 | 8.46 | 51.5 | 5 |
| I | AT2G04080 | 2 | 476 | 7.28 | 51.6 | 5 |
| I | AT2G04090 | 2 | 477 | 5.36 | 51.3 | 6 |
| I | AT2G04100 | 2 | 483 | 7.60 | 51.9 | 6 |
| I | AT2G34360 | 2 | 480 | 8.54 | 52.2 | 7 |
| I | AT3G23550 | 3 | 469 | 8.24 | 50.8 | 6 |
| I | AT3G23560 | 3 | 477 | 8.32 | 51.6 | 6 |
| I | AT5G52450 | 5 | 486 | 8.11 | 52.5 | 7 |
| II | AT1G11670 | 1 | 503 | 6.62 | 55.0 | 6 |
| II | AT1G12950 | 1 | 522 | 5.23 | 56.9 | 7 |
| II | AT1G23300 | 1 | 515 | 8.24 | 56.4 | 8 |
| II | AT1G33080 | 1 | 494 | 8.67 | 54.2 | 7 |
| II | AT1G33090 | 1 | 494 | 7.69 | 53.7 | 7 |
| II | AT1G33100 | 1 | 491 | 8.29 | 53.5 | 7 |
| II | AT1G33110 | 1 | 494 | 8.39 | 53.7 | 7 |
| II | AT1G47530 | 1 | 484 | 7.20 | 52.4 | 7 |
| II | AT1G61890 | 1 | 501 | 6.51 | 55.1 | 6 |
| II | AT3G03620 | 3 | 500 | 5.46 | 55.3 | 7 |
| II | AT3G21690 | 3 | 506 | 5.19 | 55.0 | 6 |
| II | AT3G26590 | 3 | 500 | 5.73 | 54.3 | 7 |
| II | AT3G59030 | 3 | 507 | 8.18 | 55.1 | 7 |
| II | AT4G00350 | 4 | 542 | 4.66 | 59.0 | 5 |
| II | AT4G21903 | 4 | 517 | 8.55 | 56.5 | 6 |
| II | AT4G21910 | 4 | 575 | 8.62 | 63.5 | 7 |
| II | AT4G25640 | 4 | 514 | 8.25 | 56.2 | 7 |
| II | AT5G10420 | 5 | 489 | 7.33 | 53.6 | 8 |
| II | AT5G17700 | 5 | 497 | 5.75 | 54.6 | 7 |
| II | AT5G38030 | 5 | 498 | 4.85 | 53.6 | 7 |
| II | AT5G44050 | 5 | 491 | 6.29 | 54.7 | 8 |
| II | AT5G65380 | 5 | 486 | 7.99 | 53.0 | 8 |
| III | AT1G51340 | 1 | 515 | 8.28 | 55.4 | 11 |
| III | AT2G21340 | 2 | 559 | 8.02 | 60.0 | 13 |
| III | AT2G38330 | 2 | 521 | 6.37 | 54.9 | 12 |
| III | AT3G08040 | 3 | 526 | 10.18 | 55.9 | 11 |
| III | AT4G38380 | 4 | 560 | 10.14 | 59.8 | 13 |
| III | AT4G39030 | 4 | 543 | 9.71 | 59.5 | 11 |
| IV | AT1G58340 | 1 | 532 | 8.01 | 57.8 | 1 |
| IV | AT1G71870 | 1 | 510 | 6.64 | 54.8 | 1 |
| IV | AT2G38510 | 2 | 486 | 7.40 | 52.5 | 0 |
| IV | AT4G22790 | 4 | 491 | 8.28 | 53.7 | 0 |
| IV | AT4G23030 | 4 | 502 | 7.24 | 55.2 | 0 |
| IV | AT4G29140 | 4 | 532 | 7.32 | 57.1 | 0 |
| IV | AT5G19700 | 5 | 508 | 6.60 | 54.3 | 0 |
| IV | AT5G49130 | 5 | 502 | 7.52 | 54.1 | 1 |
| IV | AT5G52050 | 5 | 505 | 7.68 | 55.0 | 0 |
