# Supplementary material for: The similar and different evolutionary trends of MATE family occurred between rice and Arabidopsis thaliana
Source: BMC Plant Biol. 2016 Sep 26;16:207. doi: 10.1186/s12870-016-0895-0 (PMC5037600; doi:10.1186/s12870-016-0895-0)
Supplement: Additional file 6: — Prediction of MATE protein members subcellular locations. (DOC 111 kb) [file 12870_2016_895_MOESM6_ESM.doc]

| **Subfamily** | **Name** | **Subcellular location** | **Reference** |
| --- | --- | --- | --- |
| MATE II | AT1G33110 | plas or vacu | - |
|  | AT1G33100 | plas | - |
|  | AT1G33090 | plas | - |
|  | AT1G33080 | plas | - |
|  | LOC_Os11g03240 | plas | - |
|  | LOC_Os12g03260 | plas | - |
|  | LOC_Os11g03500 | plas or E.R. | - |
|  | AT3G03620 | plas | - |
|  | AT5G17700 | plas | - |
|  | LOC_Os09g29284 | plas | - |
|  | LOC_Os08g37432 | cyto | - |
|  | LOC_Os03g08900 | plas | [83] |
|  | AT5G65380 | plas | - |
|  | AT5G44050 | plas | - |
|  | AT5G10420 | plas | - |
|  | AT4G00350 | plas | - |
|  | AT4G25640 | plas | - |
|  | LOC_Os08g44870 | plas | - |
|  | LOC_Os10g11860 | vacu | - |
|  | LOC_Os03g42830 | vacu | - |
|  | LOC_Os01g56050 | plas | - |
|  | LOC_Os08g43654 | plas | - |
|  | AT1G47530 | plas | - |
|  | AT1G23300 | plas | - |
|  | AT1G12950 | plas | - |
|  | AT5G38030 | vacu | - |
|  | AT3G26590 | plas or vacu | - |
|  | AT3G59030 | vacu | [16, 18] |
|  | LOC_Os12g42130 | plas | - |
|  | AT4G21903 | plas | - |
|  | AT4G21910 | plas | - |
|  | AT1G11670 | plas | - |
|  | AT1G61890 | plas | - |
|  | LOC_Os07g33310 | plas | - |
|  | AT3G21690 | plas | - |
|  | LOC_Os03g37640 | plas | - |
|  | LOC_Os03g37411 | plas | - |
|  | LOC_Os03g37490 | plas | - |
| MATE I | AT3G23550 | plas | - |
|  | AT3G23560 | plas | - |
|  | LOC_Os07g31884 | chlo | - |
|  | LOC_Os07g01750 | plas | - |
|  | LOC_Os06g29950 | plas | - |
|  | LOC_Os06g29844 | plas | - |
|  | LOC_Os06g29994 | cyto | - |
|  | LOC_Os04g30490 | plas | - |
|  | LOC_Os10g11354 | cyto | - |
|  | LOC_Os10g20350 | plas: | - |
|  | LOC_Os10g20470 | vacu: | - |
|  | LOC_Os10g20450 | plas or vacu | - |
|  | LOC_Os06g49310 | vacu | - |
|  | AT1G73700 | plas or vacu | - |
|  | AT5G52450 | plas | - |
|  | AT2G34360 | plas | - |
|  | LOC_Os01g49120 | plas | - |
|  | LOC_Os05g48040 | plas | [83] |
|  | LOC_Os01g31980 | plas | - |
|  | AT1G15150 | plas | - |
|  | AT1G15160 | plas | - |
|  | AT1G15170 | plas | - |
|  | AT1G15180 | plas | - |
|  | AT1G71140 | plas | - |
|  | AT1G64820 | plas | - |
|  | AT1G66780 | plas | - |
|  | AT1G66760 | plas | - |
|  | AT2G04090 | plas | - |
|  | AT2G04100 | plas | - |
|  | AT2G04040 | plas | - |
|  | AT2G04050 | plas | - |
|  | AT2G04080 | plas | - |
|  | AT2G04070 | plas | [18] |
| MATE IV | AT4G22790 | plas | - |
|  | LOC_Os03g64150 | plas | - |
|  | AT5G49130 | plas | - |
|  | LOC_Os12g36660 | vacu | - |
|  | AT1G71870 | cyto | - |
|  | LOC_Os03g62270 | cyto | - |
|  | AT2G38510 | plas | - |
|  | AT4G29140 | LE/PVC | [84, 85] |
|  | AT5G19700 | LE/PVC | [84] |
|  | LOC_Os06g36330 | plas | - |
|  | LOC_Os03g12790 | E.R. | - |
|  | AT1G58340 | Golg | [84, 86, 87] |
|  | AT5G52050 | plas | [84, 88] |
|  | AT4G23030 | plas | - |
|  | LOC_Os08g43250 | chlo or cyto | - |
|  | LOC_Os09g35600 | chlo or plas or E.R. | - |
|  | LOC_Os10g37920 | plas or vacu | - |
|  | LOC_Os04g48290 | vacu | - |
|  | LOC_Os02g45380 | chlo or plas | - |
| MATE III | AT4G39030 | chlo | [70, 89] |
|  | AT2G21340 | chlo | - |
|  | AT4G38380 | chlo | - |
|  | LOC_Os09g37610 | chlo | - |
|  | AT2G38330 | plas | - |
|  | LOC_Os12g01580 | chlo | - |
|  | LOC_Os03g11734 | vacu | - |
|  | LOC_Os01g69010 | plas | [37] |
|  | LOC_Os10g13940 | plas | - |
|  | AT3G08040 | vacu | - |
|  | AT1G51340 | plas | - |

The MATE protein members, which subcellular location have not been confirmed by studies, were predicted by WoLF PSORT; chlo: chloroplast envelope membrane; cyto: cytoplasm; E.R.: endoplasmic reticulum membrane; Golg: Golgi complex; vacu: vacuole membrane; LE/PVC: endosome/prevacuole; plas: Plasma membrane;
