# Supplementary figures and images for: The similar and different evolutionary trends of MATE family occurred between rice and Arabidopsis thaliana
Source: BMC Plant Biol. 2016 Sep 26;16:207. doi: 10.1186/s12870-016-0895-0 (PMC5037600; doi:10.1186/s12870-016-0895-0)

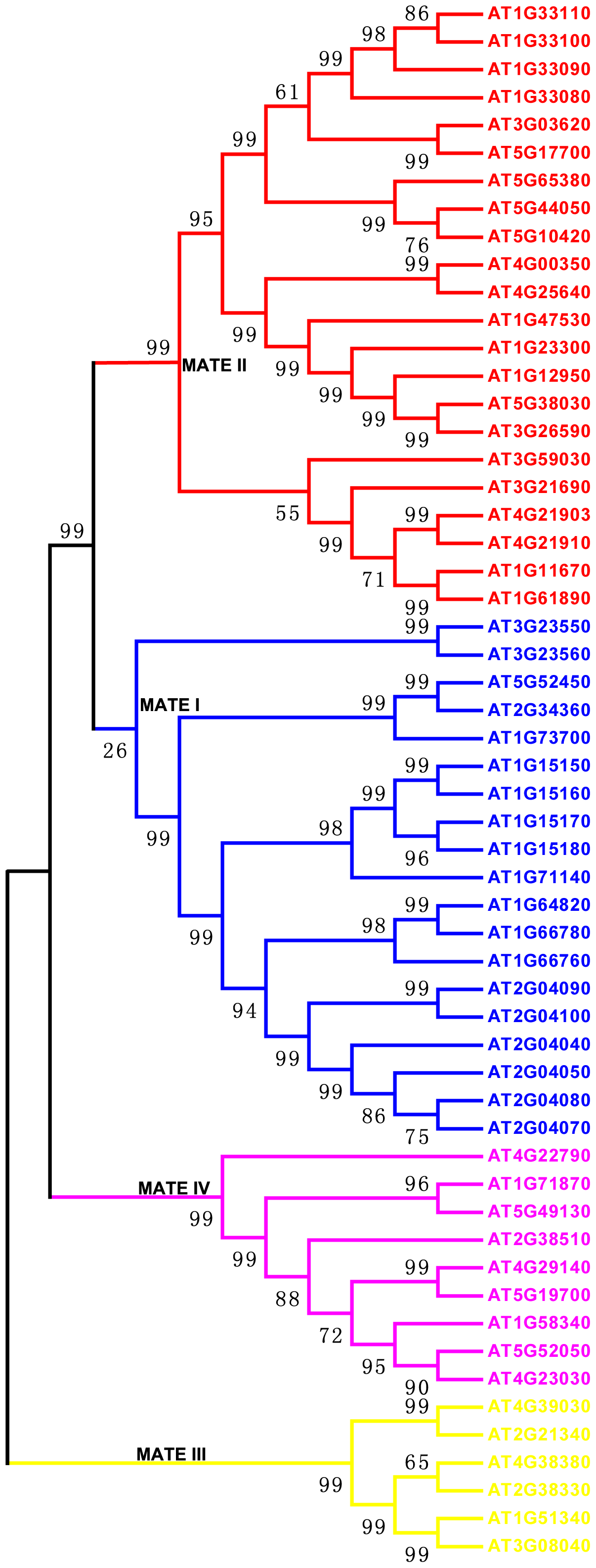

Supplement: Additional file 7: — The N-J phylogenetic tree of Arabidopsis MATE gene family. The neighbor-joining (N-J) phylogenetic tree was constructed based on a complete protein sequence alignment of 56 Arabidopsis thaliana MATE genes identified using MUSCLE and MEGA6. Numbers at the nodes represent bootstrap support values (1000 replicates). The color of subclades indicates the four corresponding gene subfamilies. (TIF 297 kb) [file 12870_2016_895_MOESM7_ESM.tif]

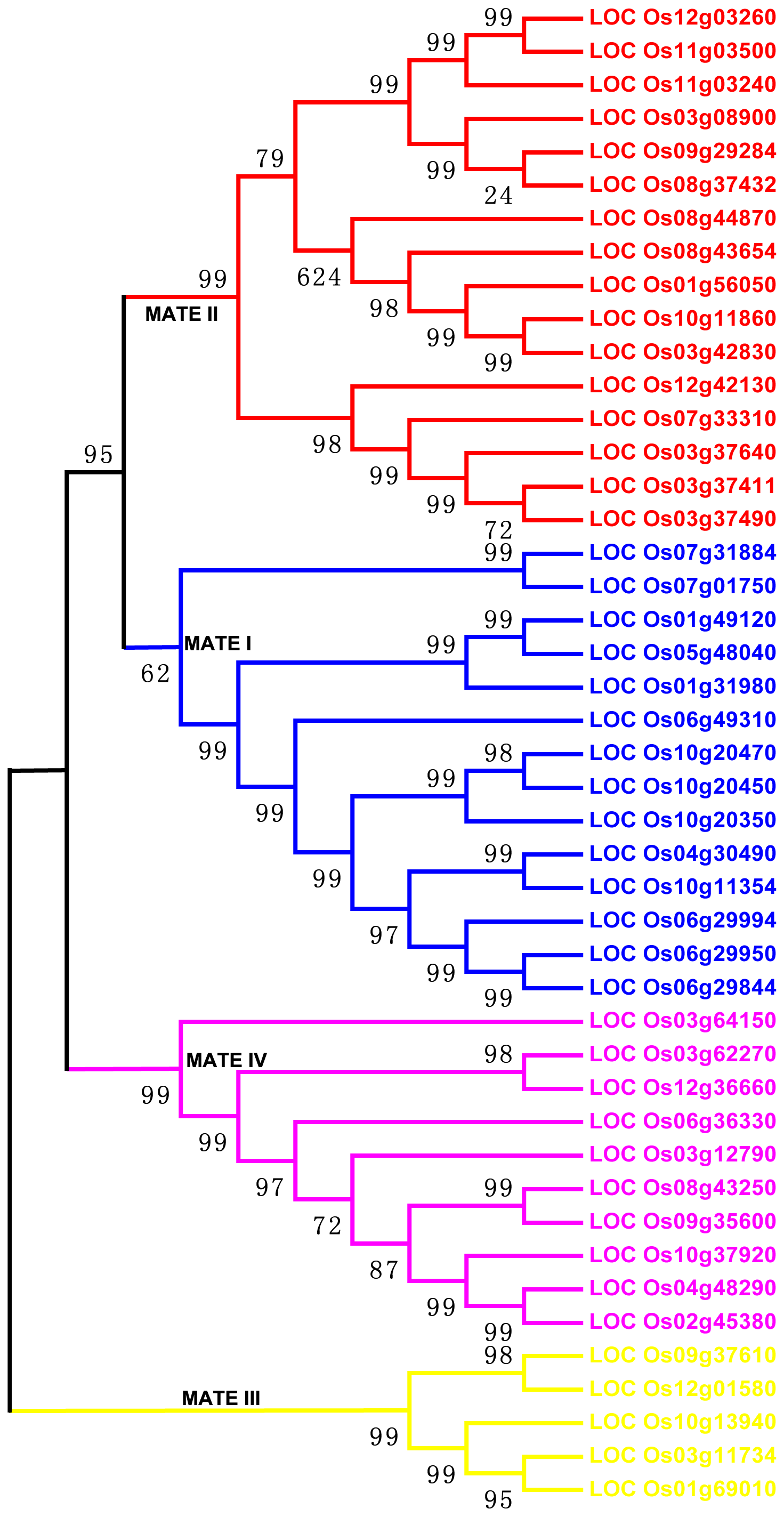

Supplement: Additional file 8: — The N-J phylogenetic tree of rice MATE gene family. The neighbor-joining (N-J) phylogenetic tree was constructed based on a complete protein sequence alignment of 45 rice MATE genes identified using MUSCLE and MEGA6. Numbers at the nodes represent bootstrap support values (1000 replicates). The color of subclades indicates the four corresponding gene subfamilies. (TIF 342 kb) [file 12870_2016_895_MOESM8_ESM.tif]

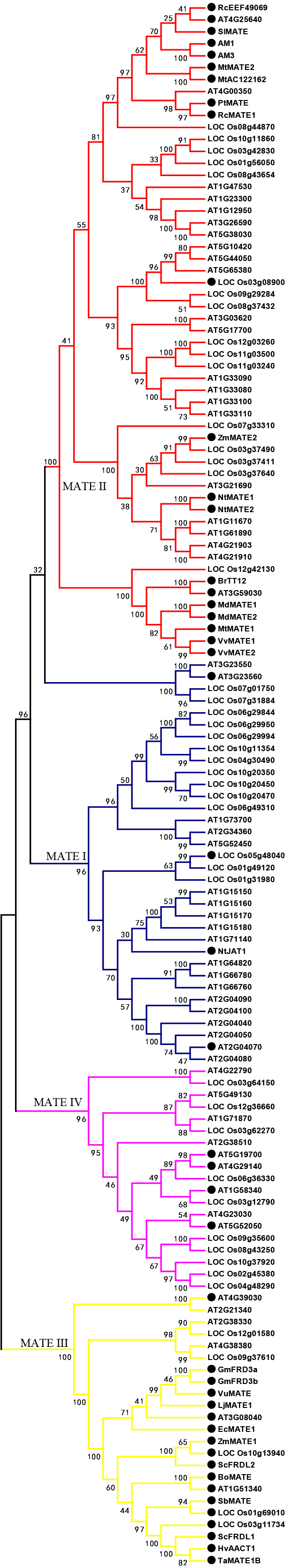

Supplement: Additional file 9: — The N-J phylogenetic tree of plant functionally known MATE menbers and 101 identified members from this study. (TIF 6275 kb) [file 12870_2016_895_MOESM9_ESM.tif]

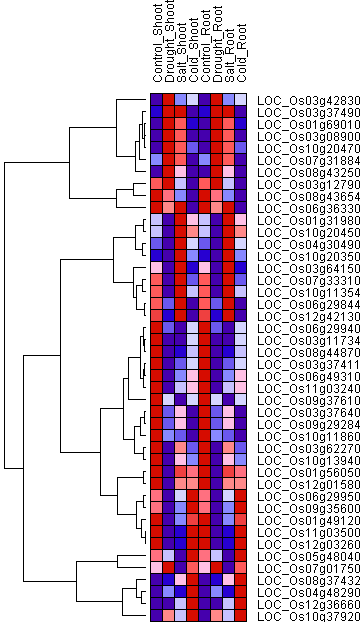

Supplement: Additional file 11: — Expression profiles of rice MATE genes under various stress. (TIFF 658 kb) [file 12870_2016_895_MOESM11_ESM.tiff]

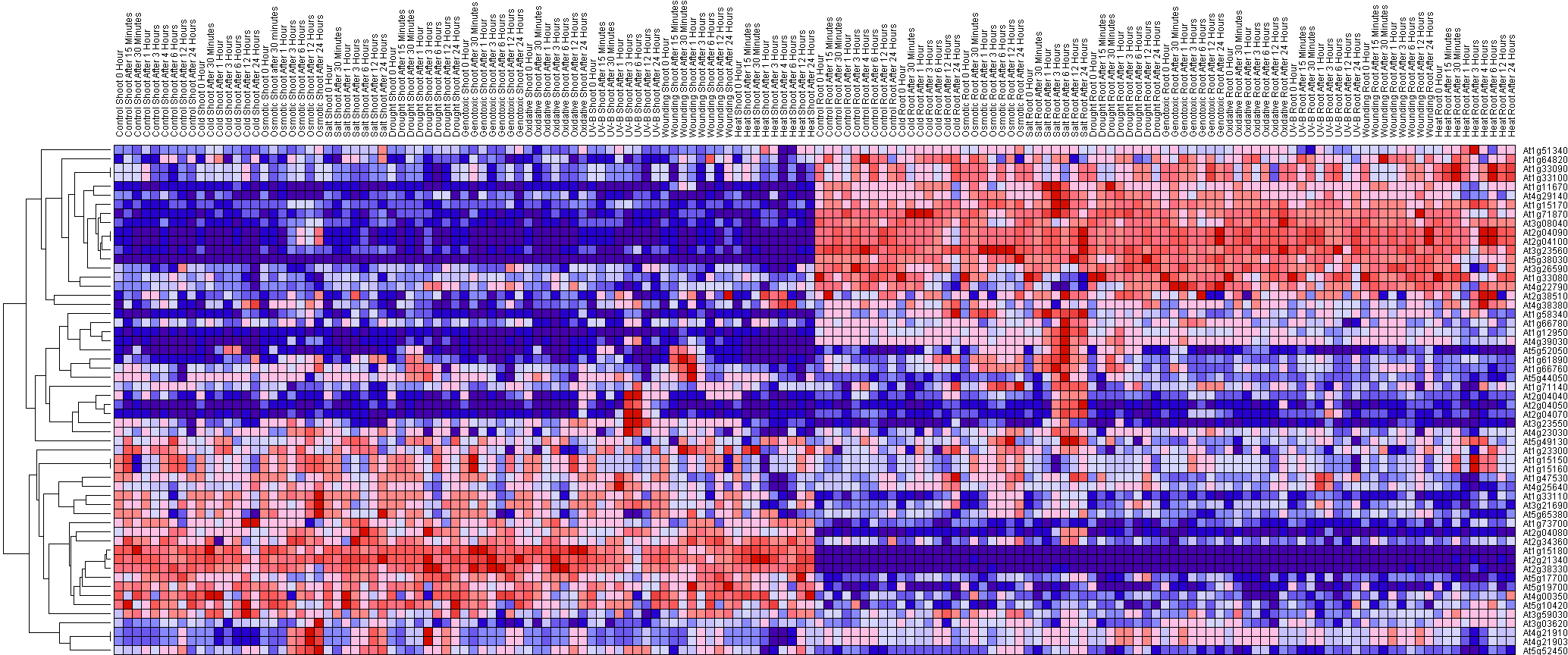

Supplement: Additional file 12: — Expression profiles of Arabidopsis MATE genes under various stress. (TIFF 5235 kb) [file 12870_2016_895_MOESM12_ESM.tiff]

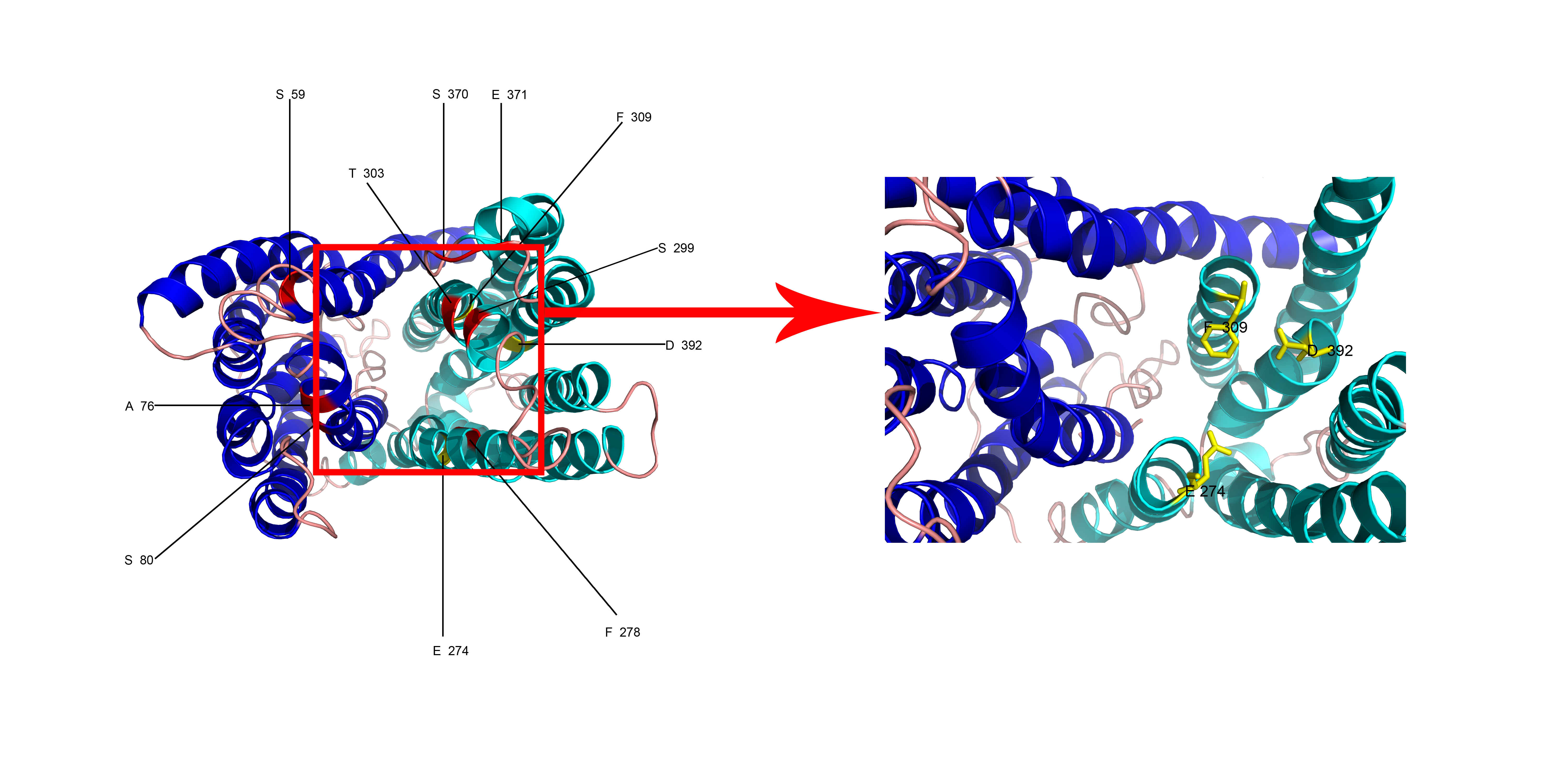

Supplement: Additional file 20: — The 3D structure of rice MATE protein LOC_Os01g49120. Amino acid sites that might interact with substrates are colored red, while those that might coordinate cation movements are colored yellow. (TIF 3471 kb) [file 12870_2016_895_MOESM20_ESM.tif]

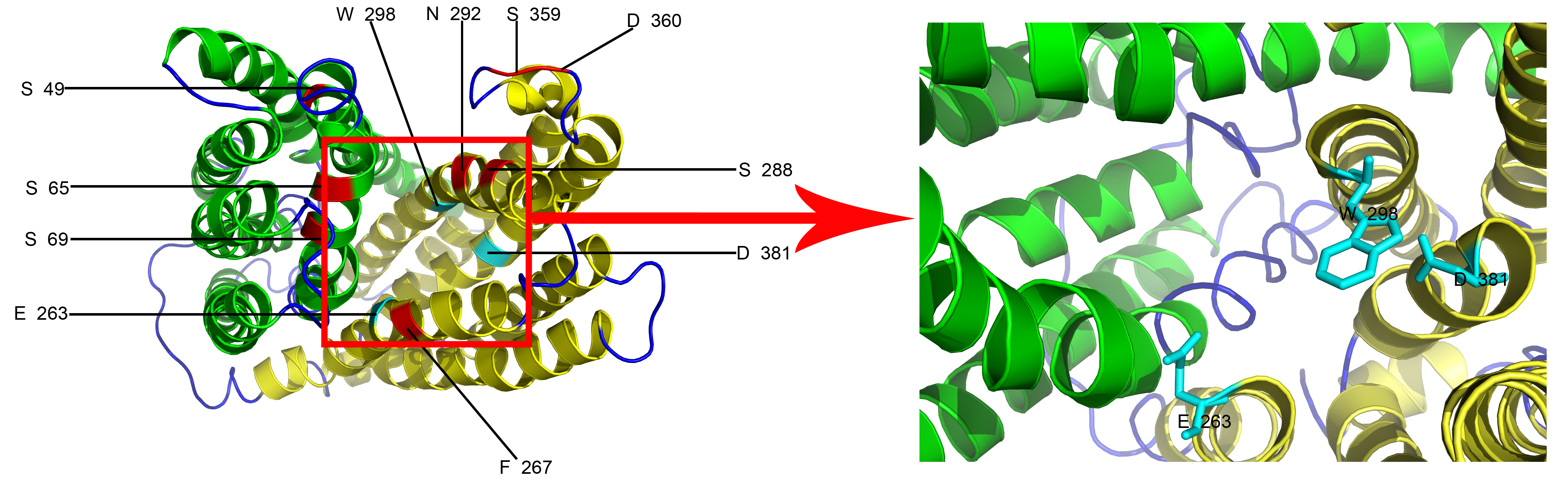

Supplement: Additional file 21: — The 3D structure of Arabidopsis thaliana MATE protein AT1G73700. Amino acid sites that might interact with substrates are colored red, while those that might coordinate cation movement are colored cyan. (TIF 3069 kb) [file 12870_2016_895_MOESM21_ESM.tif]
