# Supplementary material for: The similar and different evolutionary trends of MATE family occurred between rice and Arabidopsis thaliana
Source: BMC Plant Biol. 2016 Sep 26;16:207. doi: 10.1186/s12870-016-0895-0 (PMC5037600; doi:10.1186/s12870-016-0895-0)
Supplement: Additional file 10: — The information about plant functionally known MATE members. (DOC 98 kb) [file 12870_2016_895_MOESM10_ESM.doc]

| **Subfamily** | **Plant** | **Name** | **Subcellular localization** | **Tissue** | **Substrate** | **Proposed physiological function** | **Reference** |
| --- | --- | --- | --- | --- | --- | --- | --- |
| MATE I | Arabidopsis | ALF5  (AT3G23560) | N.D. | Root epidermis | TMA, PVP | Protection of the root from toxic compounds | [15] |
|  | Tobacco | NtJAT1 | Vacu | Root | Nicotine | Transport tobacco alkaloids from the cytosol  into the vacuole | [64] |
|  | Arabidopsis | AtDTX1  (AT2G04070) | Plas | Root | Plant-derived alkaloids, ntibiotics, and other toxic, cadmium | Efflux of plant-derived or exogenous toxic  compounds from the cytoplasm | [21] |
|  | Oryza sativa | OsMATE2  (LOC_Os05g48040) | Plas | Developing and reproductive organs | N.D. | Regulate plant growth and development as well as negatively affect disease resistance | [83] |
| MATE II | Grapevine | AM1 | Vacu | Fruit specific | Acylated anthocyanin | Mediate specifically acylated anthocyanin  transport | [90] |
|  | Grapevine | AM3 | Vacu | Fruit specific | Acylated anthocyanin | Mediate specifically acylated anthocyanin  transport | [90] |
|  | Oryza sativa | OsMATE1  (LOC_Os03g08900) | Plas | Developing and reproductive organs | N.D. | Regulate plant growth and development as well as negatively affect disease resistance | [83] |
|  | Apple | MdMATE1 | Vacu | Fruit cells | Flavonoid | Flavonoid ⁄H+-antiporters active in PA  accumulating cells | [91] |
|  | Apple | MdMATE2 | Vacu | Fruit cells | Flavonoid | Flavonoid ⁄H+-antiporters active in PA  accumulating cells | [91] |
|  | Medicago  truncatula | MtMATE1 | Vacu | Seed coat | N.D. | An essential membrane transporter for proanthocyanidin biosynthesis | [19] |
|  | Arabidopsis | TT12  (At3g59030) | Vacu | Seed coat endothelium | Flavonoids | Vacuolar flavonoid/H+ antiporter  in the seed coat | [16], [18] |
|  | Tobacco | NtMATE1 | Vacu | Root | Nicotine | Transport tobacco alkaloids from the cytosol into the vacuole | [92] |
|  | Tobacco | NtMATE2 | Vacu | Root | Nicotine | Transport tobacco alkaloids from the cytosol into the vacuole | [92] |
|  | Maize | ZmMATE2 | Plas | Not specifically localized to any particular tissue | N.D. | Suppose involved in a novel Al  tolerance mechanism | [40] |
|  | Ricinus  communis | RcEEF49069 | N.D. | N.D. | N.D. | Putative MATE, similar with TT12(At3g59030) | [93] |
|  | Arabidopsis | AtFFT  (AT4G25640) | N.D. | Floral tissues and  guard cells | Flavonoid | Flavonoid transporter | [94] |
|  | *Solanum lycopersicum* | SlMATE | Predicted Vacu | Predicted in leaves | Anthocyanin | Predicted as an anthocyanin transporter, upregulated in ANT1 transgenic line | [20] |
|  | Medicago truncatula | MtMATE2 | Vacu | Leaves and  flowers | Anthocyanins | Flavonoid transporter/vacuolar sequestration of flavonoid glycosides | [95] |
|  | Medicago truncatula | MtAC122162 | N.D. | N.D. | N.D. | Grouped together with HvAACT1 and AtFRD3 in one phylogenetic clade | [95] |
|  | Populus trichocarpa | PtMATE | N.D. | N.D. | N.D. | Similar with TT12(At3g59030) | [93] |
|  | Ricinus communis | RcMATE1 | N.D. | N.D. | N.D. | Putative MATE, similar with TT12(At3g59030) | [93] |
|  | Brassica rapa | BrTT12 | N.D. | Early and middle stages of seed  development | N.D. | Seed coat pigmentation | [96] |
|  | Vitis vinifera | VvMATE1 | Vacu | Seed development | Possible proanthocyanidin | Putative proanthocyanidin transporter | [97] |
|  | Vitis vinifera | VvMATE2 | Golg | Seed development | Possible proanthocyanidin | Putative proanthocyanidin transporter | [97] |
| MATE III | Arabidopsis | EDS5  (At4g39030) | chlo | epidermal cells | salicylic acid | transport of SA from chloroplasts to cytoplasm in epidermal cells | [70], [89] |
|  | Soybean | GmFRD3a | N.D. | N.D. | Citrate | Efflux of citrate into the root vasculature for  iron translocation | [31] |
|  | Soybean | GmFRD3b | N.D. | N.D. | Citrate | Efflux of citrate into the root vasculature for  iron translocation | [31] |
|  | Lotus japonicus | LjMATE1 | N.D. | Infection zone of nodules | Citrate | Assist the translocation of Fe from  the root to nodules | [32] |
|  | Arabidopsis | FRD3  (At3g08040) | N.D. | Root(pericycle, vascular cylinder) | Citrate | Efflux of citrate into the root vasculature for iron translocation and Zn Tolerance | [33], [34] |
|  | Eucalyptus camaldulensis | EcMATE1 | Plas | Root, shoot | Citrate | Low pH and Al-activated efflux carrier of  citrate, aluminum tolerance | [35] |
|  | Barley | HvAACT1 | Plas | Root, shoot | Citrate | Al-activated efflux carrier of citrate,  aluminum tolerance | [22] |
|  | Oryza sativa | FRDL1  (LOC_Os03g11734) | N.D. | Root pericycle | Citrate | Efficient translocation of Fe | [14] |
|  | Oryza sativa | FRDL4  (LOC_Os01g69010) | Plas | Root tip | Citrate | Al-activated efflux carrier of citrate,  aluminum tolerance | [37] |
|  | Brassica oleracea | BoMATE | Plas | Root, shoot | Citrate | Al-activated efflux carrier of citrate,  aluminum tolerance | [38] |
|  | Arabidopsis | AtMATE  (At1g51340) | N.D. | Root | Citrate | Aluminum tolerance | [39] |
|  | Maize | ZmMATE1 | Plas | Root | Citrate | Al-activated efflux carrier of citrate,  aluminum tolerance | [40] |
|  | Vigna umbellata | VuMATE | Plas | Al treatment up-regulated VuMATE expression in the root | Citrate | Al-activated efflux carrier of citrate | [41] |
|  | Rice | OsFRDL2  (LOC_Os10g13940) | unidentified vesicles in the cytosol | Root | Citrate | involved in the Al-induced secretion of citrate | [42] |
|  | Sorghum | SbMATE | Plas | Root | Citrate | aluminum resistance | [23] , [43], [44] |
|  | Secale cereale | ScFRDL1 | N.D. | Root | citrate | efflux of citrate into the xylem for Fe translocation from the roots to the shoots | [45] |
|  | Triticum aestivum | TaMATE1B | Plas | Root | citrate | Al detoxification; iron translocation | [46] |
| MATE IV | Arabidopsis | ABS3L1  (At5g19700) | LE/PVC | Young anthers, mature pollens,  developing seeds,  pistil, | N.D. | regulation of plant cell elongation | [84] |
|  | Arabidopsis | ABS3\|ADS1  (A4g29140) | LE/PVC | N.D. | N.D. | regulation of plant cell elongation; negatively  regulates plant disease resistance | [84], [85] |
|  | Arabidopsis | BCD1\|ABS4  At1g58340 | Golg | Shoot apices; stipules, mature pollen,tips of sepals | N.D. | Enhances organ initiation; Mediates homoeostasis under osmotic stress; regulation of plant cell elongation | [84], [86], [87] |
|  | Arabidopsis | DTX50\|ABS3L2  At5g52050 | Plas | Cotyledon,petioles, vascular tissues and guard cells | Abscisic acid | regulation of plant cell elongation, Efflux abscisic acid | [84], [88] |

N.D.:Not determined; Plas: Plasma membrane; LE/PVC: late endosome/prevacuole compartment;

Golg: Golgi complex; chlo: chloroplast envelope membrane; Vacu: Vacuole;

TMA: Tetramethylammonium; PVP: Polyvinylpyrrolidone
