# Supplementary material for: The similar and different evolutionary trends of MATE family occurred between rice and Arabidopsis thaliana
Source: BMC Plant Biol. 2016 Sep 26;16:207. doi: 10.1186/s12870-016-0895-0 (PMC5037600; doi:10.1186/s12870-016-0895-0)
Supplement: Additional file 13: — Type-I functional divergence sites identified between different subgroup pairs of rice and Arabidopsis. (DOC 20 kb) [file 12870_2016_895_MOESM13_ESM.doc]

| **Group1** | **Group2** | **Type-I functional divergence sites** |
| --- | --- | --- |
| OsMATE I | OsMATE II | 506M |
| OsMATE I | OsMATE III | 120S, 121D, 250T, 270M, 361W, 386Q, 399L, 502M |
| OsMATE I | OsMATE IV | 125Y, 264V, 309S, 311K, 312H, 361W |
| OsMATE II | OsMATE III | 119S, 121D, 369D, 404V, 458N |
| OsMATE II | OsMATE IV | 178S, 194L, 264V, 305L, 312H |
| OsMATE III | OsMATE IV | 119S, 121D, 216M, 218A, 264V, 271F, 322G, 484T |
| AtMATE I | AtMATE II | 72L, 93G, 142P, 249G, 264L, 279M, 397P, 454T |
| AtMATE I | AtMATE III | 86R, 108G, 149Q, 214M, 291D, 314L, 391L, 433Y |
| AtMATE I | AtMATE IV | 411I |
| AtMATE II | AtMATE III | 37E, 42W, 60I, 85I, 98A, 108G, 116G, 121R, 130T, 142P, 148G, 149Q, 156T, 164M, 180Y, 211V, 237Y, 249G, 251S, 254A, 264L, 268S, 277W, 278Y, 326N, 327E, 329G, 343I, 379V, 386L, 393N, 403A, 412V, 432T, 445G, 457L, 463K |
| AtMATE II | AtMATE IV | 42W, 85I, 120Q, 144L, 203V, 228W, 249G, 251S, 279M, 338F, 386L, 454T |
| AtMATE III | AtMATE IV | 87F, 108G, 149Q, 279M, 433Y, 452I, 456I |

**Additional file 13. The Type-I functional divergence sites identified between different subgroup pairs of Arabidopsis and rice**
