# Supplementary material for: The similar and different evolutionary trends of MATE family occurred between rice and Arabidopsis thaliana
Source: BMC Plant Biol. 2016 Sep 26;16:207. doi: 10.1186/s12870-016-0895-0 (PMC5037600; doi:10.1186/s12870-016-0895-0)
Supplement: Additional file 14: — Type-II functional divergence sites identified between different subgroup pairs of rice and Arabidopsis. (DOC 30 kb) [file 12870_2016_895_MOESM14_ESM.doc]

**Additional file 14. The Type-II functional divergence sites identified between different subgroup pairs of Arabidopsis and rice**

| **Group1** | **Group2** | **Type-II functional divergence sites** |
| --- | --- | --- |
| OsMATE I | OsMATE II | None |
| OsMATE I | OsMATE III | 104L, 105V, 107V, 108T, 109T, 110S, 111F, 112V, 113A, 114E, 119S, 120S, 121D, 123E, 124K, 125Y, 126E, 127I, 136S, 176S, 177V, 178S, 179T, 181L, 182L, 183L, 184G, 187L, 188G, 189L, 190L, 191Q, 193L, 194L, 195L, 196V, 197I, 198C, 199A, 200K, 201P, 205Y, 206M, 208V, 209K, 210Q, 211G, 212S, 213A, 214M, 215L, 216M, 217P, 219L, 220K, 221Y, 222L, 223V, 224V, 225R, 226S, 228G, 230P, 231A, 232V, 233L, 234L, 235S, 237A, 238M, 243R, 244G, 245L, 246K, 247D, 248T, 249K, 250T, 252L, 253Y, 254A, 255T, 256V, 257A, 258G, 259D, 260A, 262N, 264V, 266D, 267P, 268I, 270M, 271F, 272V, 277V, 292A, 294I, 296L, 305L, 306L, 307P, 308P, 311K, 312H, 317R, 318F, 322G, 328R, 329V, 330I, 332A, 333T, 334C, 339S, 340A, 341S, 343A, 344A, 347G, 348S, 349V, 350P, 351M, 352A, 353A, 354F, 355Q, 359Q, 360I, 361W, 362L, 363A, 364S, 365S, 366L, 367L, 368A, 369D, 372A, 373F, 375G, 376Q, 377A, 378I, 379L, 380A, 381S, 382A, 383F, 384A, 385R, 386Q, 387D, 388H, 389S, 390K, 391A, 392A, 393A, 394T, 395A, 396S, 397R, 399L, 401L, 402G, 404V, 405L, 408L, 409L, 410S, 414G,416G 417L, 418R, 420G, 421S, 422R, 423L, 424F, 425T, 426D, 427D, 428Q, 429D, 431L, 432H, 433H, 434I, 435Y, 437G, 438I, 440F, 457I, 458N, 475A, 478S, 479I, 480I, 481F, 483V, 484T, 485L, 486A, 487S, 488Y, 489N, 491F, 492V, 494I, 496I, 497A, 498L, 499T, 500V, 501Y, 502M, 503S, 504L, 505R, 508A |
| OsMATE I | OsMATE IV | None |
| OsMATE II | OsMATE III | 104L, 105V, 107V, 108T, 109T, 110S, 111F, 112V, 113A, 114E, 119S, 120S, 121D, 123E, 124K, 125Y, 126E, 127I, 136S, 176S, 177V, 178S, 179T, 180A, 181L, 183L, 184G, 187L, 188G, 189L, 191Q, 192A, 193L, 195L, 198C, 199A, 200K, 201P, 205Y, 206M, 208V, 209K, 211G, 212S, 213A, 214M, 216M, 217P, 218A, 220K, 221Y, 222L, 225R, 226S, 228G, 230P, 233L, 234L, 235S, 237A, 238M, 243R, 244G, 245L, 246K, 247D, 248T, 249K, 250T, 251P, 253Y, 254A, 255T, 256V, 258G, 259D, 260A, 262N, 266D, 267P, 268I, 270M, 271F, 272V, 277V, 278S, 292A, 295L, 296L, 305L, 306L, 307P, 308P, 311K, 312H, 317R, 318F, 322G, 327A, 328R, 330I, 333T, 334C, 339S, 341S, 343A, 344A, 347G, 348S, 350P, 351M, 352A, 354F, 355Q, 359Q, 360I, 361W, 362L, 363A, 364S, 365S, 366L, 367L, 368A, 369D, 371L, 372A, 373F, 375G, 376Q, 377A, 378I, 379L, 380A, 381S, 382A, 383F, 384A, 385R, 386Q, 387D, 388H, 389S, 390K, 391A, 392A, 393A, 394T, 395A, 396S, 397R, 399L, 401L, 402G, 405L, 408L, 409L, 410S, 412F, 414G, 417L, 418R, 419L, 420G, 421S, 422R, 427D, 428Q, 429D, 432H, 433H, 434I, 435Y, 437G, 438I, 440F, 457I, 458N, 475A, 478S, 480I, 483V, 484T, 485L, 486A, 487S, 488Y, 489N, 491F, 492V, 496I, 497A, 498L, 499T, 500V, 501Y, 502M, 503S, 505R, 506M, 508A |
| OsMATE II | OsMATE IV | None |
| OsMATE III | OsMATE IV | 105V, 106S, 107V, 108T, 109T, 110S, 111F, 112V, 113A, 114E, 119S, 120S, 121D, 123E, 124K, 125Y, 126E, 127I, 136S, 176S, 177V, 178S, 179T, 180A, 181L, 182L, 184G, 185G, 186V, 187L, 188G, 190L, 191Q, 193L, 194L, 195L, 197I, 198C, 199A, 200K, 201P, 205Y, 206M, 208V, 209K, 210Q, 211G, 212S, 213A, 214M, 215L, 216M, 217P, 218A, 219L, 220K, 221Y, 222L, 223V, 224V, 225R, 226S, 228G, 229A, 230P, 231A, 232V, 234L, 235S, 237A, 238M, 242F, 243R, 244G, 245L, 246K, 247D, 248T, 249K, 250T, 253Y, 255T, 256V, 258G, 259D, 260A, 262N, 263I, 264V, 265L, 266D, 267P, 268I, 269F, 270M, 271F, 272V, 278S, 280A, 292A, 294I, 305L, 306L, 307P, 309S, 310F, 311K, 312H, 317R, 318F, 319L, 322G, 323F, 324L, 328R, 329V, 330I, 331A, 333T, 334C, 337T, 338L, 339S, 340A, 341S, 343A, 344A, 347G, 348S, 349V, 350P, 351M, 353A, 354F, 355Q, 357C, 358L, 360I, 361W, 362L, 363A, 364S, 365S, 366L, 367L, 368A, 369D, 370G, 372A, 373F, 374A, 375G, 376Q, 377A, 378I, 379L, 380A, 381S, 382A, 383F, 384A, 385R, 386Q, 387D, 388H, 389S, 390K, 391A, 392A, 393A, 394T, 395A, 396S, 397R, 398I, 400Q, 401L, 403L, 405L, 408L, 409L, 410S, 411I, 413L, 414G, 415I, 416G, 417L, 419L, 420G, 421S, 422R, 423L, 426D, 429D, 430V, 432H, 433H, 434I, 435Y, 436L, 437G, 438I, 440F, 457I, 458N, 475A, 476I, 478S, 479I, 480I, 481F, 482I, 483V, 484T, 485L, 486A, 487S, 488Y, 489N, 492V, 494I, 496I, 497A, 499T, 500V, 501Y, 502M, 503S, 505R, 506M, 507L, 508A |
| AtMATE I | AtMATE II | None |
| AtMATE I | AtMATE III | None |
| AtMATE I | AtMATE IV | None |
| AtMATE II | AtMATE III | 35W, 36S, 37E, 40K, 43R, 46L, 51F, 52R, 54T, 55S, 56F, 57G, 58S, 62A, 71E, 80L, 81Q, 82S, 89Y, 90G, 92M, 96S, 99T, 100E, 104G, 105Q, 108G, 112Y, 113H, 114T, 118Y, 121R, 122S, 128A, 129V, 130T, 133F, 135P, 140A, 142P, 145R, 146L, 149Q, 150N, 152E, 155K, 156T, 159E, 162P, 164M, 166P, 169Y, 176T, 179M, 182Q, 184Q, 185M, 186R, 187N, 188A, 189I, 190V, 194S, 197S, 198L, 201D, 205T, 211V, 217G, 223L, 224N, 227S, 234E, 237Y, 242W, 252I, 254A, 259I, 260P, 264L, 268S, 271M, 278Y, 283V, 284L, 286A, 287G, 293K, 294I, 297S, 309E, 310L, 314L, 315G, 317L, 320A, 323R, 325A, 327E, 330K, 331G, 332D, 333A, 335A, 338F, 341K, 342V, 345T, 346I, 350M, 353I, 355S, 359L, 361F, 362C, 363G, 364R, 366S, 367Y, 371N, 373D, 377D, 380N, 381D, 382L, 383S, 384V, 388V, 390I, 391L, 394S, 396Q, 397P, 400S, 403A, 408M, 409Q, 410S, 418A, 432T, 434V, 435F, 440K, 444S, 447L, 453Q, 454T, 456I, 460I, 464T, 465D, 466W, 467E, 468L, 469E, 470V, 471K |
| AtMATE II | AtMATE IV | None |
| AtMATE III | AtMATE IV | 35W, 37E, 38V, 40K, 43R, 45A, 52R, 55S, 57G, 58S, 62A, 63Q, 71E, 76A, 77Y, 80L, 82S, 86R, 87F, 89Y, 90G, 92M, 93G, 96S, 98A, 99T, 100E, 104G, 105Q, 108G, 112Y, 114T, 121R, 122S, 128A, 132L, 133F, 134L, 139L, 140A, 146L, 149Q, 152E, 154T, 155K, 156T, 162P, 164M, 166P, 167Y, 169Y, 170S, 171L, 172I, 174T, 175M, 176T, 178Q, 179M, 184Q, 185M, 186R, 188A, 190V, 193L, 194S, 197S, 198L, 201D, 203V, 205T, 212M, 213G, 217G, 236V, 237Y, 252I, 253A, 260P, 264L, 265S, 267S, 268S, 269G, 278Y, 280S, 283V, 284L, 286A, 287G, 290K, 294I, 302C, 305I, 310L, 311N, 314L, 315G, 317L, 320A, 322V, 323R, 325A, 327E, 330K, 331G, 332D, 333A, 338F, 341K, 342V, 347S, 350M, 353I, 354F, 358C, 359L, 361F, 362C, 363G, 366S, 367Y, 371N, 373D, 374E, 379V, 380N, 381D, 382L, 384V, 387A, 388V, 390I, 391L, 392L, 394S, 395I, 396Q, 397P, 398I, 399L, 400S, 403A, 404V, 406A, 407G, 408M, 409Q, 412V, 418A, 435F, 436H, 440K, 447L, 452I, 453Q, 455I, 456I, 459Y, 460I, 464T, 465D, 466W, 467E, 468L, 469E, 470V, 471K, |
