# Supplementary material for: The similar and different evolutionary trends of MATE family occurred between rice and Arabidopsis thaliana
Source: BMC Plant Biol. 2016 Sep 26;16:207. doi: 10.1186/s12870-016-0895-0 (PMC5037600; doi:10.1186/s12870-016-0895-0)
Supplement: Additional file 15: — Tests for positive selection among codons of Arabidopsis MATE genes using site models. (DOC 19 kb) [file 12870_2016_895_MOESM15_ESM.doc]

| **Models** | ***np^a^*** | **Estimates of parameters** | **InL** | **Positively selected sites^b^** |
| --- | --- | --- | --- | --- |
| M0: one-ratio | 112 | ω =0.17742 | -52264.45 | None |
| M3: discrete | 116 | p_0_=0.28370 p_1_=0.45833 p_2_=0.25798  ω_1_ =0.06314 ω_2_=0.16810 ω_3_=0.37515 | -51477.95 | None |
| M7: beta | 113 | p =1.62137 q =6.69525 | -51450.60 | Not allowed |
| M8: beta&ɯ | 115 | p0 =0.99999 p=0.47906 q =1.60670  (p1 =0.00001 ) ω =1.16338 | -54308.40 | 35W, **43R**, **155K**, 188A, **211V**, 236V, **252I**, 259I, 360A, 364R, 434V |

Additional file 15. Tests for positive selection among codons of Arabidopsis MATE genes using site models

Note: ^a^Number of parameters in the ω distribution;

^b^Positive-selection sites are inferred at posterior probabilities > 95% with those reaching 99% shown in bold.
