# Supplementary material for: The similar and different evolutionary trends of MATE family occurred between rice and Arabidopsis thaliana
Source: BMC Plant Biol. 2016 Sep 26;16:207. doi: 10.1186/s12870-016-0895-0 (PMC5037600; doi:10.1186/s12870-016-0895-0)
Supplement: Additional file 16: — Tests for positive selection among codons of rice MATE genes using site models. (DOC 19 kb) [file 12870_2016_895_MOESM16_ESM.doc]

| **Models** | ***np^a^*** | **Estimates of parameters** | **InL** | **Positively selected sites^b^** |
| --- | --- | --- | --- | --- |
| M0: one-ratio | 90 | ω =0.18112 | -32415.328378 | None |
| M3: discrete | 94 | p_0_= 0.29456 p_1_=0.47015 p_2_= 0.23529 ω_1_ =0.05447 ω_2_=0.18269 ω_3_=0.43045 | -31784.515321 | None |
| M7: beta | 91 | p =1.36973 q =5.17116 | -31766.749156 | Not allowed |
| M8: beta&ɯ | 93 | p0 =0.99999 p=0.23865 q =1.44328  (p1 =0.00001 ) ω =1.53936 | -32817.985184 | 310F, 432H |

Additional file 16. Tests for positive selection among codons of rice MATE genes using site models

Note: ^a^Number of parameters in the ω distribution;

^b^Positive-selection sites are inferred at posterior probabilities > 95% with those reaching 99% shown in bold.
