# Supplementary material for: The similar and different evolutionary trends of MATE family occurred between rice and Arabidopsis thaliana
Source: BMC Plant Biol. 2016 Sep 26;16:207. doi: 10.1186/s12870-016-0895-0 (PMC5037600; doi:10.1186/s12870-016-0895-0)
Supplement: Additional file 17: — Critical sites identified from different rice MATE subgroups by the branch-site model. Note: aPositive-selection sites are inferred at posterior probabilities >95 % with those reaching 99 % shown in bold. (DOC 18 kb) [file 12870_2016_895_MOESM17_ESM.doc]

**Additional file 17. The critical sites identified from different rice MATE subgroups in branch-site model**

**Note: ^a^Positive-selection sites are inferred at posterior probabilities > 95% with those reaching 99% shown in bold.**

| **Cluster** | **Positive selected sites^a^** |
| --- | --- |
| OsMATE I | 235S, 433H |
| OsMATE II | **195L**, 226S, 234L |
| OsMATE III | 104L, 105V, 107V, **108T**, **109T**, **110S**, 111F, **112V**, 113A, 114E, 122R, 136S, 176S, **178S**, **179T**, **184G**, 187L, 189L 190L, **191Q**, **195L**, 198C, 200K, 206M, **208V**, **209K**, 210Q, **212S**, **214M**, **216M**, **217P**, **219L**, **221Y**, **222L**, **225R**, 226S, **228G**, **230P**, 237A, 238M, **243R**, **244G**, **245L**, **246K**, **247D**, **248T**, **249K**, **253Y**, **256V**, 258G, **259D**, 262N, **266D**, **267P**, 268I, 277V, 305L **307P**, **308P,** 312H, **317R**, 329V, **330I**, **337T**, **339S**, 340A, **341S**, **344A**, **347G**, 348S, 354F, **355Q**, **359Q**, 360I, **363A**, **365S**, **366L**, 367L, 368A, **372A**, **375G**, **376Q**, 377A, **378I**, 379L, **381S**, 383F, **384A**, **385R**, 386Q, **388H**, **390K**, 393A, 395A, **397R**, 402G, 409L, 416G, **417L**, 421S, 424F, **435Y**, **437G**, **458N**, 475A, 478S, 484T, 485L, 489N, 491F, 499T, **501Y**, 505R, 508A |
| OsMATE IV | 106S, 330I, **331A**, 357C, 370G, 376Q, 505R |
