# Supplementary material for: The similar and different evolutionary trends of MATE family occurred between rice and Arabidopsis thaliana
Source: BMC Plant Biol. 2016 Sep 26;16:207. doi: 10.1186/s12870-016-0895-0 (PMC5037600; doi:10.1186/s12870-016-0895-0)
Supplement: Additional file 18: — Critical sites identified from different Arabidopsis MATE subgroups by the branch-site model. Note: aPositive-selection sites are inferred at posterior probabilities >95 % with those reaching 99 % shown in bold. (DOC 15 kb) [file 12870_2016_895_MOESM18_ESM.doc]

**Additional file 18. The critical sites identified from different Arabidopsis MATE subgroups in branch-site model**

| **Cluster** | **Positive selected sites^a^** |
| --- | --- |
| AtMATE I | 42W, 63Q, 87F, 175M, 178Q, 205T, **350M**, **378A**, **463K** |
| AtMATE II | 38V, 48S, **55S**, 77Y, **83T**, 126D, 134L, **137I**, 150N, 160I, 161Y, **167Y**, 175M, 189I, 226G, **227S**, 234E, 251S, **276Y,** 279M, **280S**, 297S, **303Q**, 310L, 313C, 317L, 330K, 358C, **365I**, 366S, 384V, 422A |
| AtMATE III | 37E, 168V, 184Q, 186R, 292A, 404V |
| AtMATE IV | none |

**Note: ^a^Positive-selection sites are inferred at posterior probabilities > 95% with those reaching 99% shown in bold.**
