# Supplementary material for: The similar and different evolutionary trends of MATE family occurred between rice and Arabidopsis thaliana
Source: BMC Plant Biol. 2016 Sep 26;16:207. doi: 10.1186/s12870-016-0895-0 (PMC5037600; doi:10.1186/s12870-016-0895-0)
Supplement: Additional file 19: — Critical coevolving amino acids identified in rice and Arabidopsis thaliana. (DOC 11 kb) [file 12870_2016_895_MOESM19_ESM.doc]

**Additional file 19. Critical coevolving amino acids identified in rice and Arabidopsis thaliana**

| **Species** | **Coevolution amino acids** |
| --- | --- |
| *Arabidopsis thaliana* | 34V, 409Y, 454W |
| Rice | 103Q,286G, 322R, 328G, 425P |
